# Widespread Positive Selection Drives Differentiation of Centromeric Proteins in the *Drosophila melanogaster* subgroup

**DOI:** 10.1038/srep17197

**Published:** 2015-11-25

**Authors:** Emily A. Beck, Ana Llopart

**Affiliations:** 1 Interdisciplinary Graduate Program in Genetics, The University of Iowa, Iowa City, IA, 52242; 2 The Department of Biology, The University of Iowa, Iowa City, IA, 52242.

## Abstract

Rapid evolution of centromeric satellite repeats is thought to cause compensatory amino acid evolution in interacting centromere-associated kinetochore proteins. Cid, a protein that mediates kinetochore/centromere interactions, displays particularly high amino acid turnover. Rapid evolution of both Cid and centromeric satellite repeats led us to hypothesize that the apparent compensatory evolution may extend to interacting partners in the Condensin I complex (*i.e.*, SMC2, SMC4, Cap-H, Cap-D2, and Cap-G) and HP1s. Missense mutations in these proteins often result in improper centromere formation and aberrant chromosome segregation, thus selection for maintained function and coevolution among proteins of the complex is likely strong. Here, we report evidence of rapid evolution and recurrent positive selection in seven centromere-associated proteins in species of the *Drosophila melanogaster* subgroup, and further postulate that positive selection on these proteins could be a result of centromere drive and compensatory changes, with kinetochore proteins competing for optimal spindle attachment.

Rapidly evolving loci contribute to species divergence by accumulating changes themselves and by affecting interacting loci, which in turn evolve rapidly^1^,^2^,^3^. Ultimately, this rapid evolution, often identified by evidence of Darwinian positive selection to maintain function, can play an important role in speciation^2^,^3^,^4^,^5^,^6^,^7^,^8^,^9^,^10^,^11^,^12^. Fast evolution has been shown in centromeric sequences, which are comprised of rapidly evolving, tandemly repeated satellite DNAs^13^. Evolution of these repeats can drive changes in centromere-associated proteins, often resulting in coevolution through positive selection at interacting partners^1^,^14^. Specifically, this has been shown in the essential kinetochore component, H3-like histone variant, Centromere Identifier (Cid) in *Drosophila melanogaster* and *D. simulans*^1^; also called CENP-A in humans^15^,^16^.

Cid is unique in that it localizes exclusively to active centromeres during the cell cycle, and is essential for proper centromere function^17^,^18^,^19^,^20^,^21^. Cid has also been shown to play important roles in kinetochore assembly and function, and in cell-cycle progression and regulation^22^. Cid has additionally been implicated as a key player in meiotic drive, where increased recruitment of kinetochore proteins to the centromere directly results in “stronger” centromeres and preferential segregation of that chromosome to the developing oocytes^23^,^24^. The strength of the centromere, however, depends also on the genetic background. One possible source of this variation in centromere strength could be subtle differences in the sequences of Cid or other kinetochore proteins that also are part of the complex^24^. Under this scenario, one would expect to find rapid evolution not just in the satellite DNA and Cid but also possibly in all kinetochore proteins.

Mechanistically, Cid facilitates the interactions between the kinetochore and condensed chromosomes during the cell cycle^25^. This condensation is performed by the 13S condensin complex (Condensin I) composed of two structural maintenance subunits SMC2 and SMC4 (Gluon in *Drosophila*) and three non-structural maintenance subunits Cap-D2, Cap-H (Barren in *Drosophila*), and Cap-G^26^,^27^. The Condensin I complex has also been shown to interact directly with Cid via Cap-G^25^ (Fig. 1), a protein shown to bind to centromeric regions, and facilitate movement of the Condensin I complex to adjacent heterochromatin^28^. Interestingly, mutations in Cap-G result in aberrant chromosome segregation during anaphase and have little effect on chromosome condensation, suggesting an interaction between Cid and Cap-G^25^ and a link between kinetochore structure and the chromatin condensation machinery.

Cap-G has also been shown to co-localize with HP1 to the centromere^28^ (Fig. 1). HP1s are known to be important in chromosome assembly and stability^29^,^30^,^31^, and have been shown to play a role in cohesion recruitment to pericentric heterochromatin in yeast^32^. This process may result in more rigid centromeric regions which enable the chromosome to withstand the forces associated with spindle attachment, and subsequent chromosome separation^29^,^31^ without which, aberrant segregation of chromosomes could be more abundant. Additionally, it has been demonstrated that absence of essential Condensin I protein Cap-H results in abnormal centromeric heterochromatin organization, which results in a distorted centromere unable to withstand the mitotic spindle forces^33^. Previously, evidence of positive selection in one of the HP1 proteins, HP1D, also called *rhino*, was reported in the comparison of the species pair *D. melanogaster* and *D. simulans*^34^. Like HP1A, which is shown to localize exclusively to heterochromatin, HP1D and HP1E also localize to centromeric heterochromatin, although HP1D is predominantly expressed in the ovaries, while HP1E is predominantly expressed in the testes. In contrast, the expression of other HP1s in all gross adult tissues suggests differential chromatin structure in somatic *vs.* germline cells^34^.

While cases of positive selection in individual proteins associated with rapidly evolving heterochromatic regions have been reported (e.g., Cid and HP1D), it is currently unknown whether this pattern of coevolution is extended to other interacting and functionally related proteins. Here we present a comprehensive study of the entire Condensin I complex, as well as interacting proteins Cid and HP1s (including HP1A-E) in species of the *melanogaster* subgroup. These proteins work intimately with one another to confer proper spindle attachment in meiosis, without which aberrant segregation would be extensive. We analyzed 11 centromere-associated proteins in *D. melanogaster, D. simulans, D. sechellia, D. yakuba* and *D. erecta* (Fig. 2 and Supplementary Table 1). Our results confirm previous findings of positive selection in Cid and HP1D, and provide new evidence of positive selection in five additional associated proteins, including HP1C, Cap-G, Cap-D2, SMC2 and SMC4. These results of adaptive evolution detected at the level of whole protein complexes have implications for understanding meiotic drive and mechanisms of speciation.

## Results

### Evidence of adaptive evolution

To test for evidence of positive selection, we first analyzed single sequences of *D. melanogaster*, *D. simulans*, *D. sechellia, D. yakuba* and *D. erecta* for the 11 genes associated with Condensin I formation and centromere localization (Table 1). This initial assessment of positive selection was exclusively based on estimates of the number of nonsynonymous (*d*_*N*_) and synonymous (*d*_*S*_) changes per site in coding sequences and *d*_*N*_/*d*_*S*_ (*ω*) ratios in particular (see Methods). We calculated maximum likelihoods under models M1a (nearly neutral), M2a [positive selection in a fraction of the sites (ω > 1)], M7 (beta distribution), and M8 [beta distribution with positive selection in a fraction of sites (ω > 1)]. To determine whether our data fit better to models that incorporate positive selection, we then performed likelihood ratio tests comparing M1a *vs*. M2a and M7 *vs*. M8 (Table 2). The former pair is more robust for detecting positive selection^35^. When we assessed all the genes combined into a single region, we observed a significant difference between the fit of models M1a and M2a (*P* < 1 × 10^−5^) and M7 and M8 (*P* < 1 × 10^−45^) indicating a strong overall signature of positive selection in proteins associated with Condensin I formation and centromere localization (Table 2). We then evaluated each gene individually. HP1D has previously been shown to be under positive selection in multiple *Drosophila* species^34^ and we saw a significant difference between the fit of models M7 and M8 (*P* < 0.01). We did not see a significant difference in the fit of the M1a and M2a models in any other gene but did observe a significant difference in the fit of models M7 and M8 in the *cid* locus (*P* < 0.05) (Table 2). These data suggest a strong signature of adaptive evolution in *HP1D* and possibly *cid.*

**Figure 2:**
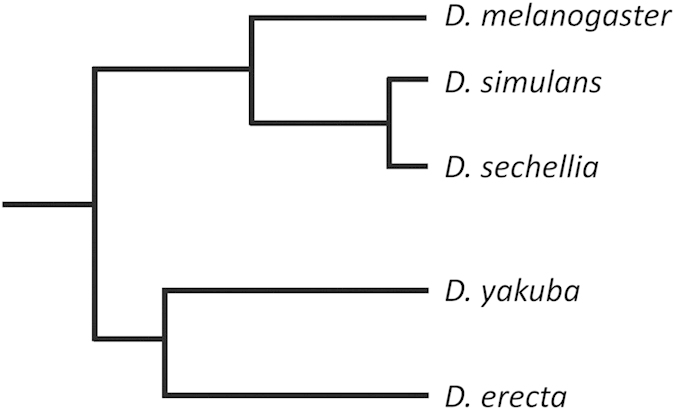
Phylogeny of *Drosophila* species analyzed in this study.

**Figure 3:**
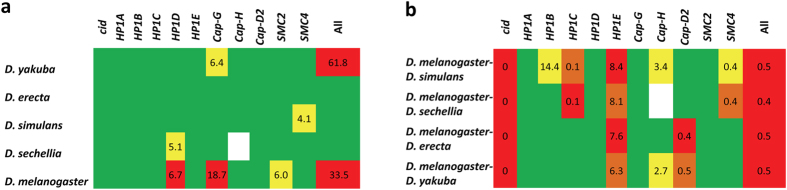
Evaluation of positive selection. (**a**) PAML assessment of positive selection in each lineage of the *D. melanogaster*, *D. simulans*, *D. sechellia*, *D. yakuba* and *D. erecta* phylogeny. The likelihood ratio test (LRT) statistic is shown within the cells. (**b**) Results of the MK test to detect positive selection based on probabilities calculated using the Fisher’s exact test. Neutrality Index (N. I.) is shown within the cells. Red: *P* < 0.01, Orange: *P* < 0.025, Yellow: *P* < 0.05, Green: *P* > 0.05, and White: Not applicable.

To determine if only one lineage or multiple lineages show trends of positive selection, we assessed the fit of our data to a model of neutrality *vs*. positive selection within each branch of the phylogeny under study (Fig. 2). We observed a significant deviation from neutrality in *HP1D* in the *D. melanogaster* (*P* = 0.009) and *D. sechellia* (*P* = 0.023) lineages, *Cap-G* in the *D. melanogaster* (*P* = 1.6 × 10^−5^) and *D. yakuba* (*P* = 0.012) lineages, *SMC2* in the *D. melanogaster* (*P* = 0.014) lineage, and *SMC4* in the *D. simulans* (*P* = 0.042) lineage (Fig. 3a and Supplementary Table 2). These results reveal evidence that proteins associated with Condensin I formation and centromere localization may often be targets of positive selection mostly, but not exclusively, along the *D. melanogaster* lineage.

To further test for positive selection and implement information from polymorphism data, we applied the McDonald-Kreitman (MK) test^36^ (see Methods). In the case of neutrality, we expect the ratio of synonymous to replacement changes to be the same for both polymorphic sites and fixed differences between species^36^,^37^. Under positive selection, however, we expect to see an increase in fixed replacement changes^36^. When we assessed the 11 loci as a group, the MK test indicated that amino acid replacements have contributed significantly and disproportionately to divergence in all comparisons (*P* < 1 × 10^−6^, Fig. 3b and Supplementary Table 3). When we assessed each gene individually, the test produced significant results in 7 of the 11 genes including *cid, HP1B, HP1C, HP1E, Cap-H, Cap-D2*, and *SMC4* (Fig. 3b and Supplementary Table 3). In four of the 7 proteins showing a significant departure from neutrality, we observe an excess of fixed replacement changes (Neutrality Index, N. I. < 1), consistent with the expectation of proteins evolving under positive selection^36^,^38^. This group excludes *HP1B*, *HP1E* and *Cap-H* that exhibit an excess of polymorphic replacements, in agreement with the potential accumulation of weakly deleterious mutations segregating within species but not reaching fixation^39^. We observe consistent signatures of positive selection across all species pairs for *cid* indicating pervasive positive selection, while the others only show evidence of positive selection in specific comparisons; *HP1C* in *D. melanogaster-D. simulans, D. melanogaster-D. sechellia*, *D. melanogaster*-*D.erecta, Cap-D2* in *D. melanogaster-D. erecta* and *D. melanogaster-D. yakuba*, and *SMC4* in *D. melanogaster-D. sechellia* and *D. melanogaster-D. simulans* (Fig. 3b and Supplementary Table 3). Note, however, that the fidelity of the MK test and its statistical power depend on the sample size, which is modest for some genes (e.g., 15 sequences analyzed in *HP1D*), due to the exclusion of *D. melanogaster* sequences with ‘Ns’ or heterozygous sites. *Cap-H* also shows a significant deficit of mutations at intermediate frequencies in *D. melanogaster* relative to the neutral expectations^40^ (*P* = 0.0096, neutral coalescent simulations with no recombination; Supplementary Table 4), consistent with an excess of replacement changes that are weakly deleterious and segregate within species as polymorphisms (see above).

### Adaptive evolution in specific protein domains

In many of the proteins analyzed, we observed a clustering of fixed replacement changes in specific domains of the proteins suggesting that some, but not all, regions of the genes may be evolving under positive selection. This is particularly apparent in *cid*, which contains a cluster of replacement changes in the N-terminal tail (Supplementary Figure 1), and *HP1C*, which shows an accumulation of replacement changes outside the conserved chromo-shadow domain (Supplementary Figure 2). To test specific regions of the proteins, we independently apply the MK test to the different protein domains in Cid, HP1A-HP1D, and Cap-G (Supplementary Table 5). These proteins were selected solely based on availability of highly characterized protein domains in *Drosophila*.

When we assessed the N-terminal tail and C-terminal core of *cid* independently, we observed a strong signature of positive selection in all lineages within the N-terminal tail consistent with the previously observed accumulation of fixed replacement changes in this region. We additionally detected positive selection in the C-terminal core in the *D. melanogaster-D. yakuba* and *D. melanogaster*-*D. erecta* species comparisons indicating that the entire protein could be evolving rapidly in these lineages. These findings are consistent with previous observations of positive selection at *cid* in the comparison between *D. melanogaster* and *D. simulans*, suggesting that centromeric proteins are undergoing fast coevolution with the interacting, ever-changing centromeric satellite repeats^1^. We also attempted to evaluate the Loop 1 DNA binding domain in *cid*, but due to a lack of polymorphisms, the MK test could not be completed. We do, however, observe a series of radical changes in all lineages that alter the charge of the protein presumably affecting DNA binding affinity and suggesting potential adaptive evolution in this region of *cid* (Supplementary Figure 1).

To assess the various protein domains of the HP1s, we analyzed the chromodomain, chromo-shadow domain, and hinge regions independently for HP1A-HP1D, as well as the C-terminal tail located outside the chromo-shadow domain in HP1C, which has an unusually long tail region. Annotations for these domains were adapted from previously published assessments of these regions^34^,^41^. As predicted based on the accumulation of fixed replacement changes in the tail region of HP1C, we observe a significant deviation from neutrality in this region alone in the *D. melanogaster-D. simulans* (*P* = 0.044) and *D. melanogaster-D. sechellia* (*P* = 0.015) species comparisons. We did not, however, identify any specific patterns of selection in the breakdown of the hinge, chromo or chromo-shadow domains, with the exception of HP1D which shows abundant replacement changes fixed between species and very low levels of polymorphism within species in all domain breakdowns. Finally, a previous study demonstrated that a C-terminal truncation of *D. melanogaster* Cap-G comprised of amino acids 1–977 was sufficient for Cap-G function and was able to rescue infertility phenotypes associated with Cap-G loss of function mutations^28^. We detected positive selection in the coding sequence corresponding to amino acids 1–977 and were unable to reject neutrality when assessing amino acids 978–1347 using the MK test (Supplementary Table 5). This suggests that the region of Cap-G primarily responsible for centromere localization and heterochromatin interaction is specifically under positive selection.

For the remaining proteins without well-characterized domains (HP1E, Cap-H, Cap-D2, SMC2, and SMC4) we performed the MK test on each exon individually except for *HP1E*, which consists of a single exon with no introns. We did not observe significant departures from neutral expectations in any exon independently with the exception of exons 2 of *Cap-D2* in the *D. melanogaster-D. erecta* species pair (*P* = 0.037; Supplementary Table 6) and exon 3 of *Cap-D2* in the D*. melanogaster*-*D. yakuba* species pair (*P* = 0.021; Supplementary Table 6). These results could indicate potential adaptive evolution in these specific regions, but more functional work is needed to determine the specific functions of the individual protein domains.

**Figure 4:**
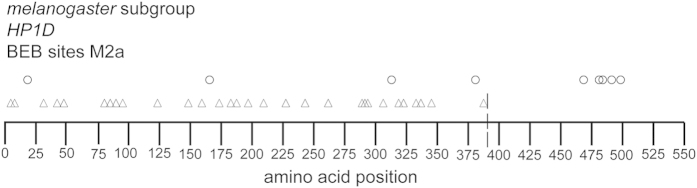
Distribution of positively selected sites in the *HP1D* gene identified using the BEB method. Triangles and circles indicate the location of positively selected and polymorphic replacement sites, respectively. The vertical dashed line represents the separation of exon 1 and exon 2.

Finally, we identified nonsynonymous sites evolving under positive selection using the BEB method in both *cid* and *HP1D*, the two genes with overall *ω* significantly greater than 1 for the entire *D. melanogaster*, *D. simulans*, *D. sechellia*, *D. yakuba* and *D. erecta* phylogeny. We observed a striking pattern of positively selected sites in *HP1D*, with all 30 positively selected sites accumulating in exon 1, with only 4 polymorphic replacements, possibly indicative of a selective sweep (Fig. 4). We also observed a clustering of polymorphisms in exon 2. This pattern was not observed in *cid*, which contained more positively selected sites in the N-terminal tail compared to the C-terminal core, but showed a fairly uniform distribution of polymorphisms throughout the coding region (Supplementary Figure 3). The distribution of positively selected sites identified by the BEB method revealed a pattern in HP1D consistent with other findings, including an accumulation of positively selected sites in exon 1 in both *D. melanogaster* and *D. sechellia* lineages (Supplementary Figures 4 and 5). We did not, however, observe distinct patterns in *Cap-G, SMC2*, or *SMC4* (Supplementary Figures 6–9).

## Discussion

Overall, our study shows that there is a considerable enrichment of adaptive evolution among proteins associated with Condensin I formation and centromere localization, with multiple signals across different species. Combined, tests of positive selection provide some evidence of adaptive evolution in the genes *cid, HP1C, HP1D, Cap-G, Cap-D2, SMC2*, and *SMC4*. These proteins interact directly with each other and, in addition, with rapidly evolving centromeric regions. Previous work showed evidence that *cid* evolves at an increased rate as a result of its physical interaction with rapidly evolving centromeric satellite repeats^1^. This protein localizes exclusively to the centromere and specifically facilitates the interactions between the kinetochore and the chromosome^25^. We also find evidence of positive selection in Cap-G, a protein known to interact physically with Cid as well as pericentric heterochromatin^25^. While the specific role of Cap-G at the centromere is not fully understood, it has been suggested that Cap-G acts as the rate limiting protein of the Condensin I complex, plays an important role in recruiting the other members of the complex to the centromere, and ultimately facilitates movement to pericentric heterochromatin^28^. Cap-G has also been shown to co-localize to the centromere with HP1s^28^, some of which are also evolving driven by positive selection. These proteins along with Cap-H, another member of the Condensin I complex that physically interacts with Cap-G, play an important role in spindle attachment, and allow the centromere to withstand mitotic spindle forces during the cell cycle^29^,^31^,^33^. The other Condensin I proteins (Cap-D2, SMC2, and SMC4) all physically interact with Cap-H, but their specific functions are unknown. All these interactions between rapidly evolving proteins that are part of multi-protein complexes would easily generate multi-locus molecular incompatibilities in hybrids and could ultimately contribute to speciation, as it has been proposed for the proteins of the nuclear pore complex^3^,^42^. More research, however, is needed to determine whether this is the case in the proteins of the Condensin I complex.

Taken together, these observations emphasize the importance of protein interactions in proper centromere assembly, function, and successful chromosome segregation during the cell cycle. While it has previously been shown that an evolutionary arms race can occur between satellite DNA and centromeric histone variants^1^, we propose that this race can extend to those proteins involved in kinetochore assembly. Our results align well with the centromere-drive hypothesis, which is based on “cheating chromosomes” that are better able to bind spindles to be preferentially incorporated into oocytes^1^. Therefore, it is possible these direct protein-protein interactions could play a role in driving a cascade of adaptive evolution as a result of a competition for optimal spindle attachment during female meiosis. Recent work in mouse supports the centromere-drive hypothesis indicating that centromere strength provides also a basis for karyotype evolution in mammals^23^,^24^. Based on these findings, it could be useful to expand this analysis to see how kinetochore-associated proteins are changing elsewhere in the tree of life.

## Methods

### Fly lines studied

Sequences of *D. melanogaster* were obtained from the *Drosophila* Genetic Reference Panel (DGRP)^43^,^44^. A list of *D. melanogaster* lines included in the analysis of each gene is shown in Supplementary Table 1. DNA sequences of *D. simulans*, *D. sechellia*, *D. yakuba* and *D. erecta* for all the genes analyzed were obtained from Flybase (http://flybase.org/)^45^ with the exception of HP1D sequences, which were obtained from NCBI accession numbers AY944331.1, AY944332.1, AY944335.1 and AY944355.1 34. Sequences containing heterozygous sites or ‘Ns’ were excluded. Due to an out-of-frame indel in the *D. sechellia* coding region of Cap-H, for this gene the *D. sechellia* sequence was removed from the analysis.

### Data analysis

To test for positive selection based on *d*_*N*_/*d*_*S*_ (ω) ratios, where *d*_*N*_ is the number nonsynonymous changes per site and *d*_*S*_ is the number synonymous changes per site, we applied the maximum likelihood method implemented in the program codeml of PAML v4.5 35. As this phylogenetic-based method does not account for within species recombination, we selected randomly a single *D. melanogaster* sequence for PAML analysis in addition to the sequences of *D. simulans*, *D. sechellia*, *D. yakuba* and *D. erecta*. We compared the fit of our data to nearly neutral models (M1a, NSites = 1 and M7 beta, NSites = 7) and to models that allow for a fraction of sites to be evolving under positive selection (M2a, NSites = 2 and M8 beta and *ω*, NSites = 8)^46^,^47^,^48^. Model M1a assumes that codons have two possible *ω* (*ω*_0_ < 1 and *ω*_1_ = 1) while M2a incorporates the possibility of an additional third class of sites under positive selection (*ω*_2_ > 1). Model M7 allows ω to vary among codons according to a beta distribution while M8 adds an additional class of codons with *ω* > 1. In all cases we used equilibrium codon frequencies calculated from the average nucleotide frequencies at third codon positions (CodonFreq = 2). Unless indicated, we assumed a single *ω* for all the lineages. When the test of positive selection significantly rejected the null hypothesis, we identified individual sites under positive selection using the Bayes empirical Bayes (BEB) calculation of posterior probabilities for site classes implemented under models M2a and M8^35^,^48^.

We additionally tested whether positive selection was occurring on each individual lineage by applying a branch-site method in which *ω* varies both among sites and among lineages (Model = 2 and NSites = 2)^49^. It is assumed that the phylogeny is divided *a priori* into foreground and background lineages and that only foreground lineages can experience positive selection. The model also assumes four different classes of codons: (1) codons conserved throughout the phylogeny, (2) codons evolving neutrally throughout the phylogeny, (3) codons conserved on the background lineages but positively selected on the foreground lineages, and (4) codons evolving neutrally on the background lineages and under positive selection on foreground lineages. The null model allows sites evolving under negative selection on the background lineages to be released from constraints and evolve neutrally on the foreground branches (fix_omega = 1 and omega = 1) while the alternative model of positive selection is constrained to a fraction of sites with *ω* > 1 on the foreground lineages (fix_omega = 0 and omega = 1.5).

To detect positive selection on protein sequences based on both polymorphism and divergence data, we also performed the McDonald-Kreitman test^36^ as implemented in DNAsp 5.1 50. This test compares levels of polymorphism at neutral and functional sites with the respective levels of divergence to determine whether neutral rates of evolution can be ruled out at functional sites. Note that we excluded *D. melanogaster* sequences with ‘Ns’ or heterozygous sites and thus the sample size and statistical power of the MK test vary among genes, ranging from 15 sequences for *HP1D* to 107 sequences for *Cap-G*. We also applied Tajima’s *D*^40^ test to evaluate whether the frequency spectrum of polymorphisms in our sample of *D. melanogaster* sequences is compatible with neutral expectations conservatively assuming no recombination.

## Additional Information

**How to cite this article**: Beck, E. A. and Llopart, A. Widespread Positive Selection Drives Differentiation of Centromeric Proteins in the *Drosophila melanogaster* subgroup. *Sci. Rep.* **5**, 17197; doi: 10.1038/srep17197 (2015).

## Supplementary Material

Supplementary Information

## Figures and Tables

**Figure 1:**
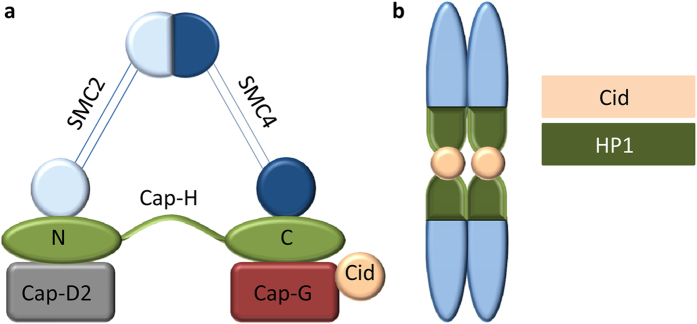
Schematic representation of the protein complexes analyzed in this study. (**a**) *Drosophila* Condensin I complex with Cid. Condensin I subunits SMC2, SMC4, Cap-H, Cap-D2, and Cap-G association with Cid. (**b**) *Drosophila* HP1 and Cid assembly at the centromere.

**Table 1:** Condensin I assembly and localization genes.

Locus in *D. melanogaster*	Length of coding region (bp)	Location in *D. melanogaster*	Cytogenetic map
*cid*	690	2R	50A11-50A11
*HP1A*	618	2L	28F2-28F3
*HP1B*	720	X	8C4-8C4
*HP1C*	714	3R	94C4-94C4
*HP1D*	1200	2R	54D2-54D2
*HP1E*	528	3R	85D11-85D11
*Cap-G*	4071	2R	49E7-49F7
*Cap-H*	2187	2L	38B1-38B2
*Cap-D2*	4125	3R	99B7-99B7
*SMC2*	3531	2R	51C5-51C5
*SMC4*	4224	2L	36A12-36A13

**Table 2:** Results of PAML comparing fit of nearly neutral (M1a and M7) and positive selection (M2a and M8) models using likelihood ratio tests (LRT).

Locus	*L*(M1a)	*L*(M2a)	LRT(M1a *vs*. M2a)	*L*(M7)	*L*(M8)	LRT(M7 *vs.* M8)
*cid*	−1799.43	−1797.64	3.59	−1800.52	−1797.35	6.33 (*P* = 0.042)
*HP1A*	−1203.97	−1203.97	0	−1203.96	−1203.96	0
*HP1B*	−1370.70	−1370.70	0	−1371.34	−1370.51	1.66
*HP1C*	−1383.14	−1383.14	0	−1383.20	−1383.20	0
*HP1D*	−3693.63	−3691.34	4.57	−3696.95	−3691.34	11.23 (*P* = 0.0037)
*HP1E*	−1298.34	−1298.34	0	−1297.50	−1297.50	0
*Cap-G*	−8882.00	−8882.00	0	−8881.92	−8881.83	0.18
*Cap-H*	−4575.88	−4575.88	0	−4574.78	−4574.78	0
*Cap-D2*	−8497.81	−8497.74	0.15	−8498.86	−8497.70	2.32
*SMC2*	−6934.41	−6934.41	0	−6933.68	−6933.65	0.048
*SMC4*	−8729.82	−8729.57	0.50	−8731.16	−8729.57	3.18
All	−66486.67	−66475.05	23.24 (*P* = 9 × 10^−6^)	−66585.83	−66477.19	217.28 (*P* = 6.6 × 10^−48^)
