# Supplementary material for: Widespread Positive Selection Drives Differentiation of Centromeric Proteins in the Drosophila melanogaster subgroup
Source: Sci Rep. 2015 Nov 25;5:17197. doi: 10.1038/srep17197 (PMC4658640; doi:10.1038/srep17197)
Supplement: Supplementary Information [file srep17197-s1.pdf]

Widespread Positive Selection Drives Differentiation of Centromeric Proteins in the *Drosophila melanogaster* subgroup

Emily A. Beck[1] and Ana Llopart[1, 2*]

[1] Interdisciplinary Graduate Program in Genetics and [2]The Department of Biology, The University of Iowa, Iowa City, IA, 52242

*Corresponding Author

Ana Llopart

469 Biology Building (BB)

The University of Iowa

Iowa City, IA, 52242

Phone: (319) 384-1880

Fax: (319) 335-1069

Email: ana-llopart@uiowa.edu

Supplementary Table 1. Lines of *D. melanogaster* used in this study.

***cid*:** 68 lines; 26, 28, 40, 41, 42, 45, 49, 57, 59, 73, 75, 83, 85, 88, 91, 105, 129, 138, 142, 153, 158, 161, 176, 181, 217, 239, 256, 310, 317, 318, 321, 332, 352, 365, 367, 371, 374, 375, 380, 383, 391, 491, 517, 555, 591, 502, 639, 703, 705, 707, 712, 714, 721, 730, 732, 738, 765, 774, 786, 804, 805, 820, 837, 852, 861, 887, 892, 897

***HP1A*:** 101 lines; 21, 26, 28, 38, 40, 41, 42, 45, 49, 59, 69, 75, 83, 91, 93, 105, 136, 149, 161, 176, 177, 195, 217, 227, 228, 229, 233, 239, 256, 280, 309, 317, 320, 321, 325, 332, 356, 358, 359, 362, 365, 367, 370, 373, 374, 379, 383, 391, 399, 406, 409, 427, 443, 461, 491, 492, 508, 509, 513, 531, 535, 555, 589, 591, 595, 646, 703, 705, 707, 712, 721, 727, 730, 732, 737, 738, 757, 761, 765, 776, 783, 786, 787, 790, 804, 808, 810, 818, 820, 837, 852, 855, 857, 859, 861, 879, 884, 887, 890, 892, 908

***HP1B*:** 77 lines; 26, 28, 38, 40, 41, 45, 57, 69, 75, 83, 91, 93, 129, 138, 149, 161, 176, 177, 181, 217, 237, 239, 280, 309, 318, 320, 321, 325, 332, 352, 359, 365, 371, 375, 377, 381, 391, 427, 443, 491, 502, 509, 513, 555, 591, 639, 642, 646, 703, 705, 707, 712, 714, 716, 730, 732, 738, 765, 776, 783, 786, 796, 799, 804, 805, 808, 810, 812, 818, 820, 837, 852, 859, 879, 882, 887, 897

***HP1C*:** 20 lines; 59, 85, 109, 161, 138, 176, 208, 233, 280, 325, 338, 356, 391, 427, 437, 491, 517, 787, 837, 887

***HP1D*:** 15 lines; 40, 88, 109, 177, 217, 321, 356, 381, 714, 732, 765, 796, 804, 810, 884

***HP1E**:** 103 lines; 21, 26, 28, 40, 42, 57, 69, 75, 85, 88, 91, 93, 101, 129, 138, 142, 149, 176, 177, 181, 208, 228, 233, 235, 239, 256, 280, 287, 310, 313, 318, 338, 357, 358, 365, 367, 370, 371, 373, **375**, 377, 379, 380, 381, 383, 391, 399, 405, 406, 409, **426**, 427, 437, 439, 441, 443, 461, 491, 492, 502, 508, 509, 513, 531, 535, 555, 589, **591**, 595, 639, 642, 646, 703, 705, 707, 714, 716, 721, 727, 730, 757, **765**, 774, 776, 783, 796, 804, 805, 810, 812, 820, 832, 837, 852, 855, 859, 861, 879, 882, 887, 892, 894, 897

***Cap-G*:** 107 lines; 26, 38, 40, 41, 42, 45, 57, 59, 69, 73, 75, 83, 85, 88, 91, 93, 105, 109, 129, 136, 138, 142, 149, 153, 158, 161, 176, 177, 181, 195, 208, 217, 227, 228, 233, 239, 256, 280, 309, 310, 318, 320, 321, 332, 352, 356, 359, 365, 367, 370, 371, 373, 374, 375, 379, 381, 383, 386, 391, 392, 399, 427, 441, 461, 491, 492, 502, 508, 513, 535, 555, 589, 591, 642, 646, 703, 705, 707, 712, 714, 716, 721, 727, 732, 737, 757, 765, 776, 783, 786, 787, 796, 799, 804, 805, 808, 810, 812, 818, 820, 837, 852, 859, 884, 887, 897, 908

***Cap-H*:** 73 lines; 21, 28, 40, 42, 57, 69, 75, 83, 91, 105, 129, 136, 138, 142, 153, 177, 181, 195, 227, 233, 239, 280, 309, 317, 320, 321, 357, 359, 362, 365, 373, 374, 375, 379, 380, 386, 391, 406, 409, 427, 437, 441, 461, 491, 508, 513, 535, 555, 639, 642, 646, 703, 714, 727, 730, 732, 737, 738, 761, 765, 783, 787, 790, 799, 805, 810, 818, 837, 882, 884, 890, 892, 908

Cap-D2**:** 59 lines; 21, 26, 42, 45, 57, 69, 73, 75, 83, 88, 93, 105, 142, 158, 161, 176, 177, 181, 235, 318, 310, 320, 325, 356, 365, 377, 381, 383, 386, 391, 399, 426, 502, 508, 509, 513, 535, 595, 703, 714, 727, 761, 765, 774, 786, 787, 796, 804, 805, 808, 810, 837, 852, 857, 859, 879, 882, 892, 894

SMC2**:** 73 lines; 21, 40, 41, 42, 57, 69, 75, 85, 91, 129, 138, 158, 177, 208, 235, 239, 256, 280, 287, 309, 313, 321, 332, 350, 358, 365, 367, 370, 371, 374, 375, 391, 392, 405, 427, 437, 440, 441, 443, 461, 491, 492, 502, 517, 531, 535, 555, 591, 595, 642, 646, 705, 712, 714, 716, 721, 732, 757, 765, 787, 790, 796, 799, 804, 808, 810, 812, 820, 837, 861, 882, 897, 907

SMC4**:** 20 lines; 26, 42, 59, 105, 181, 313, 317, 367, 375, 386, 391, 508, 591, 707, 712, 730, 732, 783, 796, 852

*Bold numbers contain a premature stop codon truncating the protein by 1 amino acid

Supplementary Table 2. Results of PAML within each lineage

Locus[a]	L(Positive Selection) [b]	L(Null) [c]	LRT statistic [d]
cid			
D. yakuba	-1794.19	-1795.38	2.38
D. erecta	-1799.19	-1799.40	0.42
D. simulans	-1799.43	-1799.43	0
D. sechellia	-1799.42	-1799.42	0
D. melanogaster	-1799.43	-1799.43	0
HP1A			
D. yakuba	-1203.97	-1203.97	0
D. erecta	-1203.97	-1203.97	0
D. simulans	-1203.92	-1203.92	0
D. sechellia	-1203.97	-1203.97	0
D. melanogaster	-1202.86	-1203.054	0.39
HP1B			
D. yakuba	-1370.70	-1370.70	0
D. erecta	-1370.70	-1370.70	0
D. simulans	-1370.70	-1370.70	0
D. sechellia	-1370.11	-1370.19	0.16
D. melanogaster	-1370.70	-1370.70	0
HP1C			
D. yakuba	-1383.14	-1383.14	0
D. erecta	-1383.058	-1383.058	0
D. simulans	-1383.14	-1383.14	0
D. sechellia	-1382.70	-1382.77	0.14
D. melanogaster	-1382.81	-1382.81	0
HP1D			
D. yakuba	-3694.76	-3696.63	3.74
D. erecta	-3696.63	-3696.63	0
D. simulans	-3696.63	-3696.63	0
D. sechellia	-3694.07	-3696.63	5.12 (*P*=0.023)
D. melanogaster	-3691.91	-3695.26	6.69 (*P*=0.009)
HP1E			
D. yakuba	-1297.98	-1298.00	0.04
D. erecta	-1297.37	-1297.37	0
D. simulans	-1298.34	-1298.34	0
D. sechellia	-1298.34	-1298.34	0
D. melanogaster	-1298.34	-1298.34	0
Cap-G			
D. yakuba	-8878.81	-8881.98	6.34 (*P*=0.012)

D. erecta	-8882.00	-8882.00	0
D. simulans	-8882.00	-8882.00	0
D. sechellia	-8881.83	-8881.83	0
D. melanogaster	-8872.54	-8881.87	18.65 (P=1.6×10^{-5})
Cap-H			
D. yakuba	-4575.69	-4575.70	0.02
D. erecta	-4568.32	-4569.57	2.51
D. simulans	-4575.88	-4575.88	0
D. sechellia	na	na	na
D. melanogaster	-4575.88	-4575.88	0
Cap-D2			
D. yakuba	-8496.75	-8497.80	2.10
D. erecta	-8495.16	-8495.21	0.01
D. simulans	-8497.81	-8497.81	0
D. sechellia	-8497.81	-8497.81	0
D. melanogaster	-8497.81	-8497.81	0
SMC2			
D. yakuba	-6934.41	-6934.41	0
D. erecta	-6933.61	-6933.61	0
D. simulans	-6934.33	-6934.33	0
D. sechellia	-6934.41	-6934.41	0
D. melanogaster	-6930.49	-6933.48	5.98 (P=0.014)
SMC4			
D. yakuba	-8729.82	-8729.82	0
D. erecta	-8722.45	-8722.88	0.86
D. simulans	-8727.67	-8729.74	4.14 (P=0.042)
D. sechellia	-8729.76	-8729.76	0
D. melanogaster	-8729.82	-8729.82	0
All			
D. yakuba	-66455.60	-66486.49	61.78 (P=3.9×10^{-15})
D. erecta	-66487.03	-66486.09	-1.89
D. simulans	-66486.49	-66486.49	0
D. sechellia	-66486.49	-66486.49	0
D. melanogaster	-66469.72	-66486.49	33.54 (P=7.0×10^{-9})

a- Gene symbols are given in *D. melanogaster*.

b- Likelihood under the positive selection model

c- Likelihood under the null model

d- LRT comparing the positive selection and null models (1 d.f.), with probabilities shown in parenthesis when $P < 0.05$

Supplementary Table 3. McDonald-Kreitman Test of Positive Selection

Locus[a]	SF[b]	SP[c]	RF[d]	RP[e]	N. I.[f]	*P*[g]
cid**						
D. melanogaster- D. simulans	20	15	22	0	NA	0.000161
D. melanogaster- D. sechellia	17	15	21	0	NA	0.000107
D. melanogaster- D. erecta	49	15	58	0	NA	0.000031
D. melanogaster- D. yakuba	45	15	68	0	NA	0.000004
HP1A						
D. melanogaster- D. simulans	16	2	7	1	1.14	ns
D. melanogaster- D. sechellia	14	2	6	1	1.17	ns
D. melanogaster- D. erecta	42	2	14	1	1.50	ns
D. melanogaster- D. yakuba	30	2	10	1	1.50	ns
HP1B						
D. melanogaster- D. simulans	18	5	1	4	14.40	0.0256
D. melanogaster- D. sechellia	13	5	2	4	5.20	ns
D. melanogaster- D. erecta	41	5	10	4	3.28	ns
D. melanogaster- D. yakuba	39	5	12	4	2.60	ns
HP1C						
D. melanogaster- D. simulans	6	14	7	1	0.06	0.0108
D. melanogaster- D. sechellia	9	14	10	1	0.06	0.0083
D. melanogaster- D. erecta	32	14	15	1	0.15	ns
D. melanogaster- D. yakuba	35	14	12	1	0.21	ns
HP1D						
D. melanogaster- D. simulans	46	4	100	6	0.69	ns
D. melanogaster- D. sechellia	43	4	107	6	0.60	ns
D. melanogaster- D. erecta	76	4	202	6	0.56	ns
D. melanogaster- D. yakuba	84	4	203	6	0.62	ns

HP1E						
D. melanogaster- D. simulans	28	2	15	9	8.40	0.0072
D. melanogaster- D. sechellia	27	2	15	9	8.10	0.0145
D. melanogaster- D. erecta	47	2	28	9	7.55	0.0079
D. melanogaster- D. yakuba	39	2	28	9	6.27	0.0208
Cap-G						
D. melanogaster- D. simulans	90	42	58	17	0.63	ns
D. melanogaster- D. sechellia	87	42	58	17	0.61	ns
D. melanogaster- D. erecta	239	42	166	17	0.58	ns
D. melanogaster- D. yakuba	214	41	141	16	0.59	ns
Cap-H						
D. melanogaster- D. simulans	57	10	17	10	3.35	0.0259
D. melanogaster- D. sechellia	-	-	-	-	-	-
D. melanogaster- D. erecta	143	10	66	10	2.17	ns
D. melanogaster- D. yakuba	146	10	55	10	2.65	0.0417
Cap-D2						
D. melanogaster- D. simulans	95	131	26	21	0.59	ns
D. melanogaster- D. sechellia	87	131	26	21	0.54	ns
D. melanogaster- D. erecta	213	131	79	21	0.43	0.00175
D. melanogaster- D. yakuba	241	131	76	21	0.51	0.0107
SMC2						
D. melanogaster- D. simulans	79	33	26	13	1.20	ns
D. melanogaster- D. sechellia	91	33	24	13	1.49	ns
D. melanogaster- D. erecta	155	33	57	13	1.07	ns
D. melanogaster- D. yakuba	190	33	47	13	1.59	ns
SMC4						
D. melanogaster- D. simulans	84	52	40	11	0.44	0.0373
D. melanogaster- D. sechellia	73	52	37	11	0.42	0.023
D. melanogaster- D. erecta	242	52	99	11	0.52	ns
D. melanogaster- D. yakuba	261	52	74	11	0.75	ns
**ALL**						
D. melanogaster- D. simulans	539	310	319	93	0.51	< 0.000001
D. melanogaster- D. sechellia	461	300	306	83	0.42	< 0.000001

D. melanogaster- D. erecta	1279	310	794	93	0.48	< 0.000001
D. melanogaster- D. yakuba	1324	309	726	92	0.54	< 0.000001

a- Gene symbols are given based on the *D. melanogaster*.

b- Synonymous changes fixed between populations

c- Synonymous polymorphisms

d- Replacement changes fixed between species

e- Replacement polymorphisms

f- Neutrality Index, with NA indicating ‘not applicable’

g- Probability based on Fisher’s Exact Test, with ns and indicating *P* > 0.05 and ‘not applicable’, respectively.

Supplementary Table 4. Tajima's *D* Test of Selection in *D. melanogaster*

Locus[a]	D[b]
cid	-0.21256
HP1A	0.03221
HP1B	1.21592
HP1C	-0.96619
HP1D	-0.85953
HP1E	0.24138
Cap-G	-1.07489
Cap-H	**-1.83663***
Cap-D2	0.29842
SMC2	-0.93946
SMC4	0.54728

a- Gene symbols are given in *D. melanogaster*

b- Tajima's D statistic

*- $P < 0.05$

Supplementary Table 5. McDonald and Kreitman test for Protein Domains

Locus[a]	SF[b]	SP[c]	RF[d]	RP[e]	*P*[f]
***cid* N-terminal Tail**					
D. melanogaster- D. simulans	9	8	18	0	0.001033
D. melanogaster- D. sechellia	9	8	16	0	0.002678
D. melanogaster- D. erecta	33	8	44	0	0.001985
D. melanogaster- D. yakuba	28	8	49	0	0.000629
***cid* C-terminal Core**					
D. melanogaster- D. simulans	11	7	4	0	ns
D. melanogaster- D. sechellia	8	7	5	0	ns
D. melanogaster- D. erecta	16	7	14	0	0.030854
D. melanogaster- D. yakuba	17	7	19	0	0.012304
***HP1A* chromodomain**					
D. melanogaster- D. simulans	1	1	0	0	NA
D. melanogaster- D. sechellia	1	1	0	0	NA
D. melanogaster- D. erecta	8	1	1	0	ns
D. melanogaster- D. yakuba	5	1	0	0	NA
***HP1A* hinge**					
D. melanogaster- D. simulans	5	0	6	1	ns
D. melanogaster- D. sechellia	5	0	5	1	ns
D. melanogaster- D. erecta	14	0	8	1	ns
D. melanogaster- D. yakuba	8	0	5	1	ns
***HP1A* chromo-shadow**					
D. melanogaster- D. simulans	4	1	0	0	NA
D. melanogaster- D. sechellia	2	1	0	0	NA
D. melanogaster- D. erecta	12	1	2	0	ns
D. melanogaster- D. yakuba	10	1	2	0	ns
***HP1B* chromodomain**					
D. melanogaster- D. simulans	4	1	0	0	NA
D. melanogaster- D. sechellia	1	1	0	0	NA
D. melanogaster- D. erecta	11	1	0	0	NA

D. melanogaster- D. yakuba	8	1	0	0	NA
***HP1B* hinge**					
D. melanogaster- D. simulans	2	1	0	0	NA
D. melanogaster- D. sechellia	2	1	1	0	ns
D. melanogaster- D. erecta	5	1	1	0	ns
D. melanogaster- D. yakuba	5	1	1	0	ns
***HP1B* chromo-shadow**					
D. melanogaster- D. simulans	6	1	0	0	NA
D. melanogaster- D. sechellia	4	1	0	0	NA
D. melanogaster- D. erecta	13	1	2	0	ns
D. melanogaster- D. yakuba	10	1	2	0	ns
***HP1C* chromodomain**					
D. melanogaster- D. simulans	0	2	0	1	NA
D. melanogaster- D. sechellia	3	2	0	1	ns
D. melanogaster- D. erecta	5	2	1	1	ns
D. melanogaster- D. yakuba	5	2	1	1	ns
***HP1C* hinge**					
D. melanogaster- D. simulans	1	1	0	0	NA
D. melanogaster- D. sechellia	1	1	0	0	NA
D. melanogaster- D. erecta	2	1	1	0	ns
D. melanogaster- D. yakuba	7	1	0	0	NA
***HP1C* chromo-shadow**					
D. melanogaster- D. simulans	1	4	2	0	ns
D. melanogaster- D. sechellia	0	4	2	0	ns
D. melanogaster- D. erecta	11	4	3	0	ns
D. melanogaster- D. yakuba	12	4	2	3	ns
***HP1C* tail**					
D. melanogaster- D. simulans	5	7	5	0	0.044118
D. melanogaster- D. sechellia	5	7	8	0	0.014654
D. melanogaster- D. erecta	14	7	10	0	ns
D. melanogaster- D. yakuba	11	7	9	0	ns

***HP1D* chromodomain**					
D. melanogaster- D. simulans	10	0	10	0	NA
D. melanogaster- D. sechellia	9	0	10	0	NA
D. melanogaster- D. erecta	13	0	16	0	NA
D. melanogaster- D. yakuba	15	0	18	0	NA
***HP1D* hinge**					
D. melanogaster- D. simulans	19	3	52	5	ns
D. melanogaster- D. sechellia	20	3	54	5	ns
D. melanogaster- D. erecta	35	3	105	5	ns
D. melanogaster- D. yakuba	39	3	110	5	ns
***HP1D* chromo-shadow**					
D. melanogaster- D. simulans	10	0	18	1	ns
D. melanogaster- D. sechellia	8	0	20	1	ns
D. melanogaster- D. erecta	8	0	25	1	ns
D. melanogaster- D. yakuba	8	0	23	1	ns
***Cap-G* 1-977**					
D. melanogaster- D. simulans	64	32	26	7	ns
D. melanogaster- D. sechellia	61	32	28	7	ns
D. melanogaster- D. erecta	174	32	97	7	0.02175
D. melanogaster- D. yakuba	148	32	83	7	0.02214
Cap-G 978-1347					
D. melanogaster- D. simulans	26	10	32	10	ns
D. melanogaster- D. sechellia	26	10	30	10	ns
D. melanogaster- D. erecta	65	10	69	10	ns
D. melanogaster- D. yakuba	66	9	58	9	ns

a- Region and species pair being assessed

b- Synonymous changes fixed between species

c- Synonymous polymorphisms

d- Replacement changes fixed between species

e- Replacement polymorphisms

f- Probability based on Fisher's Exact Test, with ns indicating $P > 0.05$ and NA indicating 'not applicable'

Supplementary Table 6. McDonald and Kreitman test for Individual Exons.

Locus[a]	SF[b]	SP[c]	RF[d]	RP[e]	*P*[f]
***Cap-H* exon 1**					
D. melanogaster- D. simulans	12	2	0	1	ns
D. melanogaster- D. sechellia	NA	NA	NA	NA	NA
D. melanogaster- D. erecta	21	2	12	1	ns
D. melanogaster- D. yakuba	22	2	7	1	ns
***Cap-H* exon 2**					
D. melanogaster- D. simulans	36	6	12	7	ns
D. melanogaster- D. sechellia	NA	NA	NA	NA	NA
D. melanogaster- D. erecta	105	6	46	7	ns
D. melanogaster- D. yakuba	101	6	39	7	ns
***Cap-H* exon 3**					
D. melanogaster- D. simulans	9	2	5	2	ns
D. melanogaster- D. sechellia	NA	NA	NA	NA	NA
D. melanogaster- D. erecta	17	2	8	2	ns
D. melanogaster- D. yakuba	23	2	9	2	ns
***Cap-D2* exon 1**					
D. melanogaster- D. simulans	3	4	0	1	ns
D. melanogaster- D. sechellia	3	4	0	1	ns
D. melanogaster- D. erecta	4	4	3	1	ns
D. melanogaster- D. yakuba	9	4	4	1	ns
***Cap-D2* exon 2**					
D. melanogaster- D. simulans	13	18	5	1	ns
D. melanogaster- D. sechellia	15	18	5	1	ns
D. melanogaster- D. erecta	22	18	11	1	0.037
D. melanogaster- D. yakuba	20	18	8	1	ns
***Cap-D2* exon 3**					
D. melanogaster- D. simulans	23	29	4	1	ns
D. melanogaster- D. sechellia	22	29	4	1	ns
D. melanogaster- D. erecta	54	29	13	1	ns

D. melanogaster- D. yakuba	62	29	18	1	0.021
***Cap-D2* exon 4**					
D. melanogaster- D. simulans	0	3	0	1	NA
D. melanogaster- D. sechellia	0	3	0	1	NA
D. melanogaster- D. erecta	6	3	1	1	ns
D. melanogaster- D. yakuba	7	3	3	1	ns
***Cap-D2* exon 5**					
D. melanogaster- D. simulans	2	2	0	0	NA
D. melanogaster- D. sechellia	2	2	0	0	NA
D. melanogaster- D. erecta	8	2	0	0	NA
D. melanogaster- D. yakuba	6	2	0	0	NA
***Cap-D2* exon 6**					
D. melanogaster- D. simulans	54	74	17	17	ns
D. melanogaster- D. sechellia	54	74	17	17	ns
D. melanogaster- D. erecta	119	74	51	17	ns
D. melanogaster- D. yakuba	137	74	43	17	ns
***SMC2* exon 1**					
D. melanogaster- D. simulans	5	1	0	0	NA
D. melanogaster- D. sechellia	7	1	0	0	NA
D. melanogaster- D. erecta	9	1	0	0	NA
D. melanogaster- D. yakuba	7	1	0	0	NA
***SMC2* exon 2**					
D. melanogaster- D. simulans	5	1	0	0	NA
D. melanogaster- D. sechellia	8	1	0	0	NA
D. melanogaster- D. erecta	8	1	1	0	ns
D. melanogaster- D. yakuba	14	1	1	0	ns
***SMC2* exon 3**					
D. melanogaster- D. simulans	22	13	4	1	ns
D. melanogaster- D. sechellia	24	13	5	1	ns
D. melanogaster- D. erecta	38	13	8	1	ns
D. melanogaster- D. yakuba	41	13	7	1	ns

***SMC2* exon 4**					
D. melanogaster- D. simulans	5	1	4	1	ns
D. melanogaster- D. sechellia	8	1	3	1	ns
D. melanogaster- D. erecta	18	1	9	1	ns
D. melanogaster- D. yakuba	24	1	6	1	ns
***SMC2* exon 5**					
D. melanogaster- D. simulans	6	3	1	2	ns
D. melanogaster- D. sechellia	5	3	0	2	ns
D. melanogaster- D. erecta	11	3	4	2	ns
D. melanogaster- D. yakuba	13	3	4	2	ns
***SMC2* exon 6**					
D. melanogaster- D. simulans	6	4	5	0	ns
D. melanogaster- D. sechellia	6	4	5	0	ns
D. melanogaster- D. erecta	11	4	2	0	ns
D. melanogaster- D. yakuba	13	4	3	0	ns
***SMC2* exon 7**					
D. melanogaster- D. simulans	29	10	12	9	ns
D. melanogaster- D. sechellia	32	10	11	9	ns
D. melanogaster- D. erecta	60	10	32	9	ns
D. melanogaster- D. yakuba	78	10	26	9	ns
***SMC4* exon 1**					
D. melanogaster- D. simulans	20	19	4	3	ns
D. melanogaster- D. sechellia	19	19	3	3	ns
D. melanogaster- D. erecta	65	19	10	3	ns
D. melanogaster- D. yakuba	58	19	11	3	ns
***SMC4* exon 2**					
D. melanogaster- D. simulans	10	9	9	2	ns
D. melanogaster- D. sechellia	11	9	8	2	ns
D. melanogaster- D. erecta	35	9	16	2	ns
D. melanogaster- D. yakuba	42	9	11	2	ns
***SMC4* exon 3**					

D. melanogaster- D. simulans	3	6	2	0	ns
D. melanogaster- D. sechellia	1	6	2	0	ns
D. melanogaster- D. erecta	17	6	6	0	ns
D. melanogaster- D. yakuba	18	6	2	0	ns
***SMC4* exon 4**					
D. melanogaster- D. simulans	37	17	14	2	ns
D. melanogaster- D. sechellia	33	17	13	2	ns
D. melanogaster- D. erecta	99	17	28	2	ns
D. melanogaster- D. yakuba	117	17	19	2	ns
***SMC4* exon 5**					
D. melanogaster- D. simulans	14	1	11	4	ns
D. melanogaster- D. sechellia	9	1	11	4	ns
D. melanogaster- D. erecta	25	1	39	4	ns
D. melanogaster- D. yakuba	25	1	31	4	ns

a- Region and species pair being assessed

b- Synonymous changes fixed between species

c- Synonymous polymorphisms

d- Replacement changes fixed between species

e- Replacement polymorphisms

f- Probability based on Fisher's Exact Test, with ns indicating *P* > 0.05 and NA indicating 'not applicable'

	6	13	14	15	16	17	18	20	27	28	29	30	35	36	37	39	40	42	43	44	51	56	57	58	60	62	63	65	72	77	78	79	80	81	86	96	107	120	123	126	129	132	147	150	152	156	164	168	173	177	181	188	193	194	195	196	197	198	200	202	204	208	214	216	217	218	219	220	221	222	224
Line714	A	A	G	C	A	G	A	C	T	G	C	A	G	G	C	G	T	G	G	C	C	A	G	T	G	C	G	A	C	C	G	G	C	C	G	G	G	C	C	A	C	C	G	T	A	C	A	G	C	G	T	G	G	C	C	G	G	A	G	A	G	C	G	G	A	G	A	G	A	C	G
Line374	.	.	.	.	.	.	.	.	.	.	.	G	.	.	.	.	.	.	.	.	.	.	.	.	.	.	.	.	.	.	.	.	.	.	.	.	.	.	.	.	.	.	.	.	.	.	.	.	.	.	.	.	.	.	.	.	.	.	.	.	.	.	.	.	.	.	.	.	.	.	.
Line321	.	.	.	.	.	.	.	.	.	.	.	.	.	.	.	.	.	.	.	.	.	.	.	.	.	.	A	.	.	.	.	.	.	.	.	.	.	.	.	.	.	.	.	.	.	.	.	.	.	.	.	.	.	.	.	.	.	.	.	.	.	.	.	.	.	.	.	.	.	.	.
Line73	.	.	.	.	.	.	.	.	C	.	.	.	.	.	.	.	.	.	.	.	.	.	.	.	.	.	.	.	.	.	.	.	.	.	.	.	.	.	.	.	.	.	.	.	.	.	.	.	.	.	.	.	.	.	.	.	.	.	.	.	.	.	.	.	.	.	.	.	.	.	.
Line730	.	.	.	.	.	.	.	.	.	.	.	.	.	.	.	.	.	.	.	.	.	.	.	.	.	.	.	.	.	.	.	.	.	.	.	.	.	.	.	.	.	.	.	.	.	.	.	.	.	.	.	.	.	.	.	.	.	.	.	.	.	.	.	.	.	.	.	.	.	.	.
Line820	.	.	.	.	.	.	.	.	.	.	.	.	.	.	.	.	.	.	.	.	.	.	.	.	.	.	.	.	.	.	.	.	.	.	.	.	.	.	.	.	.	.	.	.	.	.	.	.	.	.	.	.	.	.	.	.	.	.	.	.	.	.	.	.	.	.	.	.	.	.	.
Line42	.	.	.	.	.	.	.	.	C	.	.	.	.	.	.	.	.	.	.	.	.	.	.	.	.	.	.	.	.	.	.	.	.	.	.	.	.	.	.	.	.	.	.	.	.	.	.	.	.	.	.	.	.	.	T	.	.	.	.	.	.	.	.	.	.	.	.	.	.	.	.
Line517	.	.	.	.	.	.	.	.	C	.	.	.	.	.	.	.	.	.	.	.	.	.	.	.	.	.	.	.	.	.	.	.	.	.	.	.	.	.	.	.	.	.	.	.	.	.	.	.	.	.	.	.	.	.	T	.	.	.	.	.	.	.	.	.	.	.	.	.	.	.	.
Line105	.	.	.	.	.	.	.	.	C	.	.	.	.	.	.	.	.	.	.	.	.	.	.	.	.	.	.	.	.	.	.	.	.	.	.	.	.	.	.	.	.	.	.	.	.	.	.	.	.	.	.	.	.	.	.	.	.	.	.	.	.	.	.	.	.	.	.	.	.	.	.
Line161	.	.	.	.	.	.	.	.	.	.	.	.	.	.	.	.	.	.	.	.	.	.	.	.	.	.	.	.	.	.	.	.	.	.	.	.	.	A	.	.	.	.	.	.	.	.	.	.	.	.	.	.	.	.	.	.	.	.	.	.	.	.	.	.	.	.	.	.	.	.	.
Line732	.	.	.	.	.	.	.	.	.	.	.	.	.	.	.	.	.	.	.	.	.	.	.	.	.	.	.	.	.	.	.	.	.	.	.	.	.	A	.	.	.	.	.	.	.	.	.	.	.	.	.	.	.	.	.	.	.	.	.	.	.	.	.	.	.	.	.	.	.	.	.
Line352	.	.	.	.	.	.	.	.	.	.	.	.	.	.	.	.	.	.	.	.	.	.	.	.	.	.	.	.	.	.	.	.	.	.	.	.	.	.	.	.	.	.	.	.	.	.	.	.	.	.	.	.	.	.	.	.	.	.	.	.	.	.	.	.	.	.	.	.	.	.	.
Line712	.	.	.	.	.	.	.	.	.	.	.	.	.	.	.	.	.	.	.	.	.	.	.	.	.	.	.	.	.	.	.	.	.	.	.	.	.	.	.	.	.	.	.	.	.	.	.	.	.	.	.	.	.	.	.	.	.	.	.	.	.	.	.	.	.	.	.	.	.	.	.
Line721	.	.	.	.	.	.	.	.	.	.	.	.	.	.	.	.	.	.	.	.	.	.	.	.	.	.	.	.	.	.	.	.	.	.	.	.	.	.	.	.	.	.	.	.	.	.	.	.	.	.	.	.	.	.	.	.	.	.	.	.	.	.	.	.	.	.	.	.	.	.	.
Line26	.	.	.	.	.	.	.	.	C	.	.	.	.	.	.	.	.	.	.	.	.	.	.	.	.	.	.	.	.	.	.	.	.	.	.	.	.	.	.	.	.	.	A	.	.	.	.	.	.	.	.	.	.	.	.	.	.	.	.	.	.	.	.	.	.	.	.	.	.	.	.
Line138	.	.	.	.	.	.	.	.	C	.	.	.	.	.	.	.	.	.	.	.	.	.	.	.	.	.	.	.	.	.	.	.	.	.	.	.	.	.	.	.	.	.	.	.	.	.	.	.	.	.	.	.	.	.	T	.	.	.	.	.	.	.	.	.	.	.	.	.	.	.	.
Line181	.	.	.	.	.	.	.	.	C	.	.	.	.	.	.	.	.	.	.	.	.	.	.	.	.	.	.	.	.	.	.	.	.	.	.	.	.	.	.	.	.	.	.	.	.	.	.	.	.	.	.	.	.	.	T	.	.	.	.	.	.	.	.	.	.	.	.	.	.	.	.
Line367	.	.	.	.	.	.	.	.	C	.	.	.	.	.	.	.	.	.	.	.	.	.	.	.	.	.	.	.	.	.	.	.	.	.	.	.	.	.	.	.	.	.	.	.	.	.	.	.	.	.	.	.	.	.	T	.	.	.	.	.	.	.	.	.	.	.	.	.	.	.	.
Line375	.	.	.	.	.	.	.	.	C	.	.	.	.	.	.	.	.	.	.	.	.	.	.	.	.	.	.	.	.	.	.	.	.	.	.	.	.	.	.	.	.	.	.	.	.	.	.	.	.	.	.	.	.	.	T	.	.	.	.	.	.	.	.	.	.	.	.	.	.	.	.
Line786	.	.	.	.	.	.	.	.	C	.	.	.	.	.	.	.	.	.	.	.	.	.	.	.	.	.	.	.	.	.	.	.	.	.	.	.	.	.	.	.	.	.	.	.	.	.	.	.	.	.	.	.	.	.	T	.	.	.	.	.	.	.	.	.	.	.	.	.	.	.	.
sechelia	.	.	.	T	.	.	.	T	C	.	.	.	.	.	.	.	.	.	.	.	.	.	.	.	.	.	.	.	T	.	A	.	.	.	.	.	.	.	.	G	.	.	.	.	.	A	.	.	.	.	A	.	.	.	.	.	.	.	.	.	.	T	A	.	.	A	T	.	G	T	.
simulans	.	.	.	T	.	.	.	.	C	.	.	.	.	.	.	.	.	.	.	.	.	.	A	.	.	.	.	.	.	.	.	.	.	.	A	.	.	.	.	G	.	A	.	.	.	A	.	.	.	.	A	.	.	.	.	.	.	.	.	.	.	T	A	.	.	A	T	.	G	T	.
erecta	C	.	A	T	G	C	T	.	.	A	A	C	A	C	.	A	.	.	A	T	.	.	.	C	T	.	A	G	.	A	A	.	.	A	.	.	A	.	G	G	.	.	.	A	.	G	.	T	G	C	A	-	A	T	.	A	.	C	A	C	A	.	A	C	T	A	C	A	C	T	.
yakuba	.	G	.	G	.	A	C	.	C	.	G	C	A	A	T	.	A	.	.	T	T	G	.	C	.	A	A	C	.	.	A	T	G	.	.	A	.	.	G	.	T	.	.	A	G	G	G	T	.	.	A	A	.	.	.	.	A	.	.	.	.	.	.	.	.	A	T	A	G	G	C
														R																																																									
	S	R	R	S	R	R	S	R	S	R	R	S	R	S	R	S	R	S	R	R	S	R	S	R	S	R	S	R	S	R	S	R	R	S	R	S	R	S	S	S	S	S	S	S	R	R	R	S	R	S	R	R	R	R	S	R	R	S	R	S	S	R	R	S	R	R	R	R	R	S	R
												P																																																											
	F	F	F	F	F	F	F	F	P	F	F	F	F	F	F	F	F	F	F	F	F	F	F	F	F	F	P	F	F	F	F	F	F	F	F	F	F	P	F	F	F	F	P	F	F	F	F	F	F	F	F	F	F	F	P	F	F	F	F	F	F	F	F	F	F	F	F	F	F	F	F

	228	229	234	236	243	252	258	265	266	270	271	272	274	277	278	279	280	282	283	284	289	291	296	297	298	299	300	301	303	304	308	309	311	312	317	318	320	321	322	327	330	331	333	334	336	340	345	348	351	363	371	372	375	379	380	382	393	399	400	405	406	408	409	412	416	417	423
Line714	C	A	T	G	G	C	G	T	A	C	A	C	A	A	G	A	T	G	C	C	A	G	G	A	A	T	G	A	C	G	A	G	A	G	G	C	A	A	A	A	A	G	A	G	G	G	T	G	A	G	G	G	C	G	C	G	G	A	G	G	A	A	A	G	G	T	C
Line374	.	.	.	.	.	.	.	.	.	.	.	.	.	.	.	.	.	.	.	.	.	.	.	.	.	.	.	.	.	.	.	.	.	.	.	.	.	.	.	.	.	.	.	.	.	.	.	.	.	.	.	.	.	.	.	.	.	.	.	.	.	.	.	.	.	.	.
Line321	.	.	.	.	.	.	.	.	.	.	.	.	.	.	.	.	.	.	.	.	.	.	.	.	.	.	.	.	.	.	.	.	.	.	.	.	.	.	.	.	.	.	.	.	.	.	.	.	.	.	.	.	.	.	.	.	.	.	.	.	.	.	.	.	.	.	.
Line73	.	.	.	.	.	.	.	.	.	.	.	.	.	.	.	.	.	.	.	.	.	.	.	.	.	.	.	.	.	.	.	.	.	.	.	.	.	.	.	.	.	.	.	.	.	.	.	.	.	.	.	.	.	.	.	.	.	.	.	.	.	.	.	.	.	.	.
Line730	.	.	.	.	.	.	.	.	.	.	.	.	.	.	.	.	.	.	.	.	.	.	.	.	.	.	.	.	.	.	.	.	.	.	.	.	.	.	.	.	.	.	.	.	.	.	.	.	.	.	.	.	.	.	.	.	.	.	.	.	.	.	.	.	.	.	.
Line820	.	.	.	.	.	.	.	.	.	.	.	.	.	.	.	.	.	.	.	.	.	.	.	.	.	.	.	.	.	.	.	.	.	.	.	.	.	.	.	.	.	.	.	.	.	.	.	.	.	.	.	.	.	.	.	.	.	.	.	.	.	.	.	.	.	.	.
Line42	.	.	.	.	.	.	.	.	.	.	.	.	.	.	.	.	.	.	.	.	.	.	.	.	.	.	.	.	.	.	.	.	.	.	.	.	.	.	.	.	.	.	.	.	.	.	.	.	.	.	.	.	.	.	.	.	.	.	.	.	.	.	.	.	.	.	.
Line517	.	.	.	.	.	.	.	.	.	.	.	.	.	.	.	.	.	.	.	.	.	.	.	.	.	.	.	.	.	.	.	.	.	.	.	.	.	.	.	.	.	.	.	.	.	.	.	.	.	.	.	.	.	.	.	.	.	.	.	.	.	.	.	.	.	.	.
Line105	.	.	.	.	.	.	.	.	.	.	.	.	.	.	.	.	.	.	.	.	.	.	.	.	.	.	.	.	.	.	.	.	.	.	.	.	.	.	.	.	.	.	.	.	.	.	.	.	.	.	.	.	.	.	.	.	.	.	.	.	.	.	.	.	.	.	.
Line161	.	.	.	.	.	.	.	.	.	.	.	.	.	.	.	.	.	.	.	.	.	.	.	.	.	.	.	.	.	.	.	.	.	.	.	.	.	.	.	.	.	.	.	.	.	.	.	.	.	.	.	.	.	.	.	.	.	.	.	.	.	.	.	.	.	.	.
Line732	.	.	.	.	.	.	.	.	.	.	.	.	.	.	.	.	.	.	.	.	.	.	.	.	.	.	.	.	.	.	.	.	.	.	.	.	.	.	.	.	.	.	.	.	.	.	.	.	.	.	.	.	.	.	.	.	.	.	.	.	.	.	.	.	.	.	.
Line352	.	.	.	.	.	.	.	.	.	.	.	.	.	.	.	.	.	.	.	.	.	.	.	.	.	.	.	.	.	.	.	.	.	.	.	.	.	.	.	.	.	.	.	.	.	.	.	.	.	.	.	.	.	.	.	.	.	.	.	.	.	.	.	.	.	.	.
Line712	.	.	.	.	.	.	.	.	.	.	.	.	.	.	.	.	.	.	.	.	.	.	.	.	.	.	.	.	.	.	.	.	.	.	.	.	.	.	.	.	.	.	.	.	.	.	.	.	.	.	.	.	.	.	.	.	.	.	.	.	.	.	.	.	.	.	.
Line721	.	.	.	.	.	.	.	.	.	.	.	.	.	.	.	.	.	.	.	.	.	.	.	.	.	.	.	.	.	.	.	.	.	.	.	.	.	.	.	.	.	.	.	.	.	.	.	.	.	.	.	.	.	.	.	.	.	.	.	.	.	.	.	.	.	.	.
Line26	.	.	.	.	.	.	.	.	.	.	.	.	.	.	.	.	.	.	.	.	.	T	.	.	.	.	.	.	.	.	.	.	.	.	.	.	.	.	.	.	.	.	.	.	.	.	.	.	.	.	.	.	.	.	.	.	.	.	.	.	.	.	.	.	.	.	.
Line138	.	.	.	.	.	.	.	.	.	.	.	.	.	.	.	.	.	.	.	.	.	.	.	.	.	.	.	.	.	.	.	.	.	.	.	.	.	.	.	.	.	.	.	.	.	.	.	.	.	.	.	.	G	.	.	.	.	.	.	.	.	.	.	.	.	.	.
Line181	.	.	.	.	.	.	.	.	.	.	.	.	.	.	.	.	.	.	.	.	.	.	.	.	.	.	.	.	.	.	.	.	.	.	.	.	.	.	.	.	.	.	.	.	.	.	.	.	.	.	.	.	G	.	.	.	.	.	.	.	.	.	.	.	.	.	.
Line367	.	.	.	.	.	.	.	.	.	.	.	.	.	.	.	.	.	.	.	.	.	.	.	.	.	.	.	.	.	.	.	.	.	.	.	.	.	.	.	.	.	.	.	.	.	.	.	.	.	.	.	.	G	.	.	.	.	.	.	.	.	.	.	.	.	.	.
Line375	.	.	.	.	.	.	.	.	.	.	.	.	.	.	.	.	.	.	.	.	.	.	.	.	.	.	.	.	.	.	.	.	.	.	.	.	.	.	.	.	.	.	.	.	.	.	.	.	.	.	.	.	G	.	.	.	.	.	.	.	.	.	.	.	.	.	.
Line786	.	.	.	.	.	.	.	.	.	.	.	.	.	.	.	.	.	.	.	.	.	.	.	.	.	.	.	.	.	.	.	.	.	.	.	.	.	.	.	.	.	.	.	.	.	.	.	.	.	.	.	.	G	.	.	.	.	.	.	.	.	.	.	.	.	.	.
sechelia	.	G	.	.	A	.	.	C	.	.	.	G	G	.	.	.	C	.	T	.	.	.	.	.	T	.	.	.	.	.	.	.	.	.	.	.	.	G	.	G	.	.	.	.	.	.	.	.	G	.	.	.	.	.	.	.	.	.	.	.	.	G	.	.	.	C	.
simulans	A	G	.	.	.	.	.	C	.	.	.	G	G	.	.	.	C	.	T	.	.	.	.	.	T	.	.	.	.	A	.	.	.	.	.	.	.	G	.	G	.	.	.	.	.	.	.	.	G	.	.	.	.	.	.	A	.	.	.	.	.	G	.	.	.	C	T
erecta	-	-	C	A	.	.	.	C	.	G	G	.	G	.	.	.	.	A	A	A	.	.	C	G	.	C	A	C	A	.	G	C	.	A	C	T	G	C	T	G	.	.	G	.	A	T	.	A	G	.	.	.	.	A	T	.	C	.	.	.	C	G	.	.	T	C	A
yakuba	.	.	C	A	T	T	A	G	G	G	G	.	G	G	C	G	.	.	A	.	T	.	.	G	.	.	.	.	.	.	.	C	C	A	C	.	G	C	.	G	G	C	G	A	A	.	C	A	G	A	A	A	.	A	T	C	A	G	A	T	.	G	T	A	.	C	A
					R																											R						R																													
	S	R	S	R	S	S	S	R	R	S	R	R	R	R	R	S	R	S	R	R	R	S	R	S	R	R	S	R	S	R	R	S	R	S	R	S	R	S	R	S	S	R	S	R	S	R	S	S	S	S	R	S	S	R	R	R	R	S	R	R	S	S	R	R	R	S	S
	F	F	F	F	F	F	F	F	F	F	F	F	F	F	F	F	F	F	F	F	F	P	F	F	F	F	F	F	F	F	F	R	F	F	F	F	F	F	F	F	F	F	F	F	F	F	F	F	F	F	F	F	P	F	F	F	F	F	F	F	F	F	F	F	F	F	F

	425	429	435	436	442	447	453	466	471	472	480	486	492	496	510	512	513	515	516	520	522	529	534	541	543	544	546	555	561	567	572	574	588	589	590	591	610	611	624	627	651	652	664	666	675	676	677	682	683	684	685	686	687
Line714	G	A	G	C	C	C	G	C	C	T	G	C	C	G	C	A	C	A	G	C	A	A	G	C	T	T	G	G	G	G	A	T	C	G	C	G	A	A	C	C	A	T	A	C	G	G	G	C	A	A	T	T	T
Line374	.	.	.	.	.	.	.	.	.	.	.	.	.	.	.	.	.	.	.	.	.	.	.	.	.	.	.	.	.	.	.	.	.	.	.	.	.	.	.	.	.	.	.	.	.	.	.	.	.	.	.	.	.
Line321	.	.	.	.	.	.	.	.	.	.	.	.	.	.	.	.	.	.	.	.	.	.	.	.	.	.	.	.	.	.	.	.	.	.	.	.	.	.	.	.	.	.	.	.	.	.	.	.	.	.	.	.	.
Line73	.	.	.	.	.	.	.	.	.	.	.	.	.	.	.	.	.	.	.	.	.	.	.	.	.	.	.	.	.	.	.	.	.	.	.	.	.	.	.	.	.	.	.	.	.	.	.	.	.	.	.	.	.
Line730	.	.	.	.	.	.	.	.	.	.	A	.	.	.	.	.	.	.	.	.	.	.	A	.	A	.	.	.	.	.	.	.	.	.	.	C	.	.	.	.	.	.	.	.	.	.	.	.	.	.	.	.	.
Line820	.	.	.	.	.	.	.	.	.	.	A	.	.	.	.	.	.	.	.	.	.	.	A	.	A	.	.	.	.	.	.	.	.	.	.	C	.	.	.	.	.	.	.	.	.	.	.	.	.	.	.	.	.
Line42	.	.	.	.	.	.	.	.	.	.	A	.	.	.	.	.	.	.	.	.	.	.	A	.	A	.	.	.	.	.	.	.	.	.	.	.	.	.	.	.	.	.	.	.	.	.	.	.	.	.	.	.	.
Line517	.	.	.	.	.	.	.	.	.	.	A	.	.	.	.	.	.	.	.	.	.	.	A	.	A	.	.	.	.	.	.	.	.	.	.	.	.	.	.	.	.	.	.	.	.	.	.	.	.	.	.	.	.
Line105	.	.	.	.	.	.	.	.	.	.	A	.	.	.	.	.	.	.	.	.	.	.	A	.	A	.	.	.	.	.	.	.	.	.	.	.	.	.	.	.	.	.	.	.	.	.	.	.	.	.	.	.	.
Line161	.	.	.	.	.	.	.	.	.	.	A	.	.	.	.	.	.	.	.	.	.	.	A	.	A	.	.	.	.	.	.	.	.	.	.	.	.	.	.	.	.	.	.	.	.	.	.	.	.	.	.	.	.
Line732	.	.	.	.	.	.	.	.	.	.	A	.	.	.	.	.	.	.	.	.	.	.	A	.	A	.	.	.	.	.	.	.	.	.	.	.	.	.	.	.	.	.	.	.	.	.	.	.	.	.	.	.	.
Line352	.	.	.	.	.	.	.	.	.	.	A	.	.	.	.	.	.	.	.	.	.	.	A	.	A	.	.	.	.	.	.	.	.	.	.	.	.	.	.	.	.	.	.	.	.	.	.	.	.	.	.	.	.
Line712	.	.	.	.	.	.	.	.	.	.	A	.	.	.	.	.	.	.	.	.	.	.	A	.	A	.	.	.	.	.	.	.	.	.	.	.	.	.	.	.	.	.	.	.	.	.	.	.	.	.	.	.	.
Line721	.	.	.	.	.	.	.	.	.	.	A	.	.	.	.	.	.	.	.	.	.	.	A	.	A	.	.	.	.	.	.	.	.	.	.	.	.	.	.	.	.	.	.	.	.	.	.	.	.	.	.	.	.
Line26	.	.	A	.	.	.	.	.	.	.	.	.	.	.	.	.	.	.	.	.	.	.	A	.	A	.	.	.	.	.	.	.	.	.	.	.	.	.	.	.	.	.	.	.	.	.	.	.	.	.	.	.	.
Line138	.	.	.	.	.	.	.	.	.	.	.	.	.	.	.	.	.	.	.	.	.	.	.	.	A	.	.	.	.	.	.	.	.	.	.	C	.	.	.	.	.	.	.	.	.	.	.	.	.	.	.	.	C
Line181	.	.	.	.	.	.	.	.	.	.	.	.	.	.	.	.	.	.	.	.	.	.	.	.	A	.	.	.	.	.	.	.	.	.	.	C	.	.	.	.	.	.	.	.	.	.	.	.	.	.	.	.	C
Line367	.	.	.	.	.	.	.	.	.	.	.	.	.	.	.	.	.	.	.	.	.	.	.	.	A	.	.	.	.	.	.	.	.	.	.	C	.	.	.	.	.	.	.	.	.	.	.	.	.	.	.	.	C
Line375	.	.	.	.	.	.	.	.	.	.	.	.	.	.	.	.	.	.	.	.	.	.	.	.	A	.	.	.	.	.	.	.	.	.	.	C	.	.	.	.	.	.	.	.	.	.	.	.	.	.	.	.	C
Line786	.	.	.	.	.	.	.	.	.	.	.	.	.	.	.	.	.	.	.	.	.	.	.	.	A	.	.	.	.	.	.	.	.	.	.	C	.	.	.	.	.	.	.	.	.	.	.	.	.	.	.	.	C
sechelia	.	.	.	.	.	.	.	.	.	.	.	G	T	A	.	G	.	.	.	.	G	.	.	.	A	A	.	A	C	A	.	.	.	.	.	.	.	.	.	.	.	.	.	.	.	.	.	.	.	.	G	C	.
simulans	.	.	.	.	.	T	.	.	.	.	.	G	T	A	.	G	.	.	.	.	G	.	.	.	A	.	A	A	C	A	.	.	.	.	.	.	.	.	.	.	.	.	.	.	.	.	.	.	.	.	G	C	.
erecta	.	.	.	A	T	.	.	.	.	.	.	.	.	.	T	G	A	.	.	T	G	T	.	T	.	A	.	.	C	.	.	G	G	T	.	.	C	G	.	G	T	.	C	T	.	.	.	.	T	G	G	C	C
yakuba	T	G	.	A	.	.	T	A	T	G	.	.	.	.	T	.	A	C	A	T	G	.	.	T	A	A	T	.	C	.	T	.	.	.	A	.	.	.	A	.	C	C	.	.	A	A	A	T	C	G	.	C	A
	R	S	S	R	R	S	S	R	S	R	S	S	S	R	S	R	S	R	S	S	S	R	S	R	S	R	R<br>S	S	S	S	R	R	S	R	R	S	S	R	S	S	S	S	R	S	S	R	R	R	R	S	R	R	S
	F	F	P	F	F	F	F	F	F	F	P	F	F	F	F	F	F	F	F	F	F	F	P	F	P	F	F	F	F	F	F	F	F	F	F	P	F	F	F	F	F	F	F	F	F	F	F	F	F	F	F	F	P<br>F

Supplementary Figure 1. Characterization of variable sites in *cid.* Polymorphisms in 20 strains of *D. melanogaster* and one strain of *D. sechellia*, *D. simulans, D. erecta,* and *D. yakuba.* Nucleotide position (N position) is given. Synonymous vs. Replacement changes are indicated (S) and (R). Fixed differences and polymorphisms are indicated (F) and (P). Some sites are designated as both S and R if different changes occurred in different lineages. The Replacement changes in these cases are shaded grey. The N-terminal tail and C-terminal core domains are separated by an additional space.

a)

	33	54	63	72	80	93	96	102	123	126	141	162	174	177
Line85	G	T	A	T	A	T	C	G	G	C	T	A	G	C
Line517	.	.	.	.	.	.	.	.	.	.	.	.	.	.
Line59	.	.	.	.	.	.	.	.	.	.	.	.	.	.
Line161	.	.	.	.	.	.	.	.	.	.	.	.	.	.
Line176	.	.	.	.	.	.	.	.	.	.	.	.	.	.
Line233	.	.	.	.	.	.	.	.	.	.	.	.	.	.
Line280	.	.	.	.	.	.	.	.	.	.	.	.	.	.
Line338	.	.	.	.	.	.	.	.	.	.	.	.	.	.
Line356	.	.	.	.	.	.	.	.	.	.	.	.	.	.
Line391	.	.	.	.	.	.	.	.	.	.	.	.	.	.
Line427	.	.	.	.	.	.	.	.	.	.	.	.	.	.
Line437	.	.	.	.	.	.	.	.	.	.	.	.	.	.
Line491	.	.	.	.	.	.	.	.	.	.	.	.	.	.
Line787	.	.	.	.	.	.	.	.	.	.	.	.	.	.
Line887	.	.	.	.	.	.	.	.	.	.	.	.	.	.
Line138	.	.	.	.	.	.	.	.	.	.	.	.	.	.
Line208	.	.	.	.	.	.	.	.	.	.	.	.	.	.
Line325	.	.	.	.	.	.	.	.	.	.	.	.	.	.
Line837	.	C	.	G	T	.	.	.	.	.	.	.	.	.
Line109	.	C	.	G	T	.	.	.	.	.	.	.	.	.
sechelia	.	C	.	G	T	.	A	.	A	.	.	.	T	.
simulans	.	C	.	G	T	.	.	.	.	.	.	.	.	.
yakuba	.	C	C	G	.	C	.	.	A	G	.	G	.	T
erecta	A	C	C	G	.	C	.	C	.	.	C	.	.	T
	S	S	R	S	R	S	S	S	S	S	S	S	S	S
	F	P	F	P	P	F	F	F	F	F	F	F	F	F

	186	192	207	210	216	219	225	228	232	234
Line85	C	G	A	C	C	A	C	G	C	G
Line517	.	.	.	.	.	.	.	.	.	.
Line59	.	.	.	.	.	.	.	.	.	.
Line161	.	.	.	.	.	.	.	.	.	.
Line176	.	.	.	.	.	.	.	.	.	.
Line233	.	.	.	.	.	.	.	.	.	.
Line280	.	.	.	.	.	.	.	.	.	.
Line338	.	.	.	.	.	.	.	.	.	.
Line356	.	.	.	.	.	.	.	.	.	.
Line391	.	.	.	.	.	.	.	.	.	.
Line427	.	.	.	.	.	.	.	.	.	.
Line437	.	.	.	.	.	.	.	.	.	.
Line491	.	.	.	.	.	.	.	.	.	.
Line787	.	.	.	.	.	.	.	.	.	.
Line887	.	.	.	.	.	.	.	.	.	.
Line138	.	.	.	.	.	.	.	.	.	.
Line208	.	.	.	.	.	.	.	.	.	.
Line325	.	.	.	.	.	.	.	.	.	.
Line837	.	.	.	.	.	.	.	.	.	.
Line109	.	.	.	.	.	G	.	.	.	.
sechelia	.	.	.	A	.	G	.	.	.	.
simulans	.	.	.	.	.	G	.	.	.	.
yakuba	A	A	G	.	T	G	T	A	.	.
erecta	A	.	G	.	.	G	.	.	A	.
	S	S	S	S	S	S	S	S	R	S
	F	F	F	F	F	P	F	F	F	F

	237	246	253	279	282	294	306	312	321	323	330	340	351	354	360	362	376	389	390	393	402	408	411
Line85	C	G	T	A	G	C	T	G	G	T	C	T	G	A	C	T	C	A	C	T	G	A	T
Line517	.	.	.	.	.	.	.	.	.	.	.	.	.	.	.	.	.	.	.	.	.	.	.
Line59	.	.	.	.	.	.	.	.	.	.	.	.	.	.	.	.	.	.	.	.	.	.	.
Line161	.	.	.	.	.	.	.	.	.	.	.	.	.	.	.	.	.	.	.	.	.	.	.
Line176	.	.	.	.	.	.	.	.	.	.	.	.	.	.	.	.	.	.	.	.	.	.	.
Line233	.	.	.	.	.	.	.	.	.	.	.	.	.	.	.	.	.	.	.	.	.	.	.
Line280	.	.	.	.	.	.	.	.	.	.	.	.	.	.	.	.	.	.	.	.	.	.	.
Line338	.	.	.	.	.	.	.	.	.	.	.	.	.	.	.	.	.	.	.	.	.	.	.
Line356	.	.	.	.	.	.	.	.	.	.	.	.	.	.	.	.	.	.	.	.	.	.	.
Line391	.	.	.	.	.	.	.	.	.	.	.	.	.	.	.	.	.	.	.	.	.	.	.
Line427	.	.	.	.	.	.	.	.	.	.	.	.	.	.	.	.	.	.	.	.	.	.	.
Line437	.	.	.	.	.	.	.	.	.	.	.	.	.	.	.	.	.	.	.	.	.	.	.
Line491	.	.	.	.	.	.	.	.	.	.	.	.	.	.	.	.	.	.	.	.	.	.	.
Line787	.	.	.	.	.	.	.	.	.	.	.	.	.	.	.	.	.	.	.	.	.	.	.
Line887	.	.	.	.	.	.	.	.	.	.	.	.	.	.	.	.	.	.	.	.	.	.	.
Line138	.	.	.	.	.	.	.	.	.	.	.	.	.	.	.	.	.	.	.	.	.	.	.
Line208	.	.	.	.	.	.	.	.	.	.	.	.	.	.	.	.	.	.	.	.	.	.	.
Line325	.	.	.	.	.	.	.	.	.	.	.	.	.	.	.	.	.	.	.	.	.	.	.
Line837	.	.	.	.	.	.	.	.	.	.	.	.	.	.	.	.	.	.	.	.	.	.	.
Line109	.	.	C	C	.	.	.	.	.	.	.	.	.	.	.	.	.	.	.	.	.	G	G
sechelia	.	.	C	C	.	.	.	.	.	.	.	.	.	.	.	.	A	.	A	.	.	G	.
simulans	.	.	C	C	.	T	.	.	.	.	.	.	.	.	.	.	A	.	A	.	.	G	.
yakuba	T	A	C	C	A	.	C	.	A	.	T	C	A	T	A	C	.	C	.	C	.	G	C
erecta	T	A	C	C	.	.	C	T	A	A	.	.	.	T	A	C	.	C	A	C	A	G	C
	S	S	S	S	S	S	S	S	S	R	S	S	S	S	S	R	R	R	S	S	S	S	S
	F	F	P	P	F	F	F	F	F	F	F	F	F	F	F	F	F	F	F	F	F	P	P

b)

	420	429	431	432	463	468	471	480	494	495	504	513	516	525	528	535	549	558	564
Line85	T	G	T	G	T	G	C	C	T	T	G	A	G	G	G	T	T	T	A
Line517	.	.	.	.	.	.	.	.	.	.	.	.	.	.	.	.	.	.	.
Line59	.	.	.	.	.	.	.	.	.	.	.	.	.	.	.	.	.	.	.
Line161	.	.	.	.	.	.	.	.	.	.	.	.	.	.	.	.	.	.	.
Line176	.	.	.	.	.	.	.	.	.	.	.	.	.	.	.	.	.	.	.
Line233	.	.	.	.	.	.	.	.	.	.	.	.	.	.	.	.	.	.	.
Line280	.	.	.	.	.	.	.	.	.	.	.	.	.	.	.	.	.	.	.
Line338	.	.	.	.	.	.	.	.	.	.	.	.	.	.	.	.	.	.	.
Line356	.	.	.	.	.	.	.	.	.	.	.	.	.	.	.	.	.	.	.
Line391	.	.	.	.	.	.	.	.	.	.	.	.	.	.	.	.	.	.	.
Line427	.	.	.	.	.	.	.	.	.	.	.	.	.	.	.	.	.	.	.
Line437	.	.	.	.	.	.	.	.	.	.	.	.	.	.	.	.	.	.	.
Line491	.	.	.	.	.	.	.	.	.	.	.	.	.	.	.	.	.	.	.
Line787	.	.	.	.	.	.	.	.	.	.	.	.	.	.	.	.	.	.	.
Line887	.	.	.	.	.	.	.	.	.	.	.	.	.	.	.	.	.	.	.
Line138	.	.	.	.	.	.	.	.	.	.	.	.	.	.	.	.	.	C	.
Line208	.	.	.	.	.	.	.	A	.	A	.	.	.	.	C	.	.	.	.
Line325	.	.	.	.	.	.	.	A	.	A	.	.	.	.	C	.	.	.	.
Line837	.	.	.	.	.	.	.	A	.	A	.	.	.	.	C	.	.	.	.
Line109	.	.	.	.	.	.	.	.	.	.	.	.	.	.	.	.	.	C	.
sechelia	.	A	.	.	.	.	.	.	C	.	.	.	.	.	.	G	.	C	.
simulans	.	A	.	.	.	.	.	.	C	.	.	.	.	.	C	G	.	C	.
yakuba	G	.	A	C	C	.	.	.	.	A	.	G	.	T	.	G	.	C	T
erecta	G	.	G	C	.	A	A	.	.	C	T	C	T	.	.	G	C	C	T
												R							
	S	S	R	R	S	S	S	S	R	S	S	S	S	R	S	R	S	S	S
	F	F	F	F	F	F	F	P	F	P	F	F	F	F	P	F	F	P	F

	567	573	579	590	591	604	606	616	618	622	624	628	634	637	642	645	663	710	711
Line85	C	A	G	C	G	G	A	C	A	A	T	A	G	T	T	T	C	A	T
Line517	.	.	.	.	.	.	.	.	.	.	.	.	.	.	.	.	.	.	.
Line59	.	.	.	.	.	.	.	.	.	.	.	.	.	.	.	.	.	.	.
Line161	.	.	.	.	.	.	.	.	.	.	.	.	.	.	.	.	.	.	.
Line176	.	.	.	.	.	.	.	.	.	.	.	.	.	.	.	.	.	.	.
Line233	.	.	.	.	.	.	.	.	.	.	.	.	.	.	.	.	.	.	.
Line280	.	.	.	.	.	.	.	.	.	.	.	.	.	.	.	.	.	.	.
Line338	.	.	.	.	.	.	.	.	.	.	.	.	.	.	.	.	.	.	.
Line356	.	.	.	.	.	.	.	.	.	.	.	.	.	.	.	.	.	.	.
Line391	.	.	.	.	.	.	.	.	.	.	.	.	.	.	.	.	.	.	.
Line427	.	.	.	.	.	.	.	.	.	.	.	.	.	.	.	.	.	.	.
Line437	.	.	.	.	.	.	.	.	.	.	.	.	.	.	.	.	.	.	.
Line491	.	.	.	.	.	.	.	.	.	.	.	.	.	.	.	.	.	.	.
Line787	.	.	.	.	.	.	.	.	.	.	.	.	.	.	.	.	.	.	.
Line887	.	.	.	.	.	.	.	.	.	.	.	.	.	.	.	.	.	.	.
Line138	.	G	.	.	.	.	G	.	.	.	.	.	.	.	.	.	.	.	.
Line208	.	G	.	.	A	.	G	.	.	.	.	.	.	.	.	.	.	.	.
Line325	.	G	.	.	A	.	G	.	.	.	.	.	.	.	.	.	.	.	.
Line837	.	G	.	.	A	.	G	.	.	.	.	.	.	.	.	.	.	.	.
Line109	.	G	.	.	.	.	G	.	.	.	.	.	.	.	.	.	.	.	.
sechelia	G	G	.	T	.	C	G	A	.	G	C	.	A	.	C	C	A	.	.
simulans	G	G	.	T	.	.	G	.	.	G	C	.	.	.	C	C	A	.	.
yakuba	G	.	A	T	.	A	G	.	C	G	.	C	.	C	C	C	A	.	C
erecta	G	C	.	T	.	A	G	.	C	G	.	C	.	C	C	C	A	C	.
	R	S	S	R	S	R	S	R	S	R	S	R	R	S	S	S	S	R	S
	F	P	F	F	P	F	P	F	F	F	F	F	F	F	F	F	F	F	F

Supplementary Figure 2. Characterization of variable sites in *HP1C.* Polymorphisms within 20 strains of *D. melanogaster* and 1 strain of *D. sechellia*, *D. simulans, D. erecta,* and *D. yakuba.* Nucleotide position (N position) is given. Synonymous vs. Replacement changes are indicated (S) and (R). Fixed differences and polymorphisms are indicated (F) and (P). Some sites are designated as both S and R if different changes occurred in different lineages. The Replacement changes in these cases are shaded grey. **a**) Chromodomain, hinge, and chromoshadow domains separated by spaces. **b**) The tail region after the chromoshadow domain.

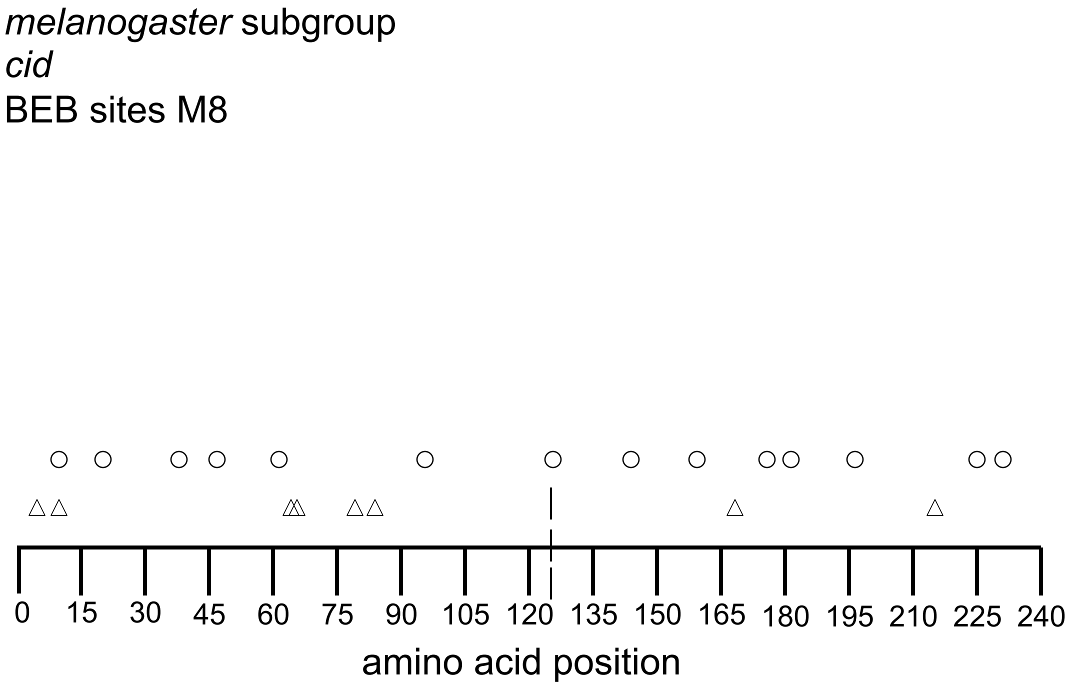

Supplementary Figure 3. Distribution of positively selected sites in the *cid* gene identified using the BEB method. Triangles indicate the location of positively selected sites, circles indicate the location of polymorphisms. The vertical dashed line represents the separation of the N-terminal tail from the C-terminal core**.**

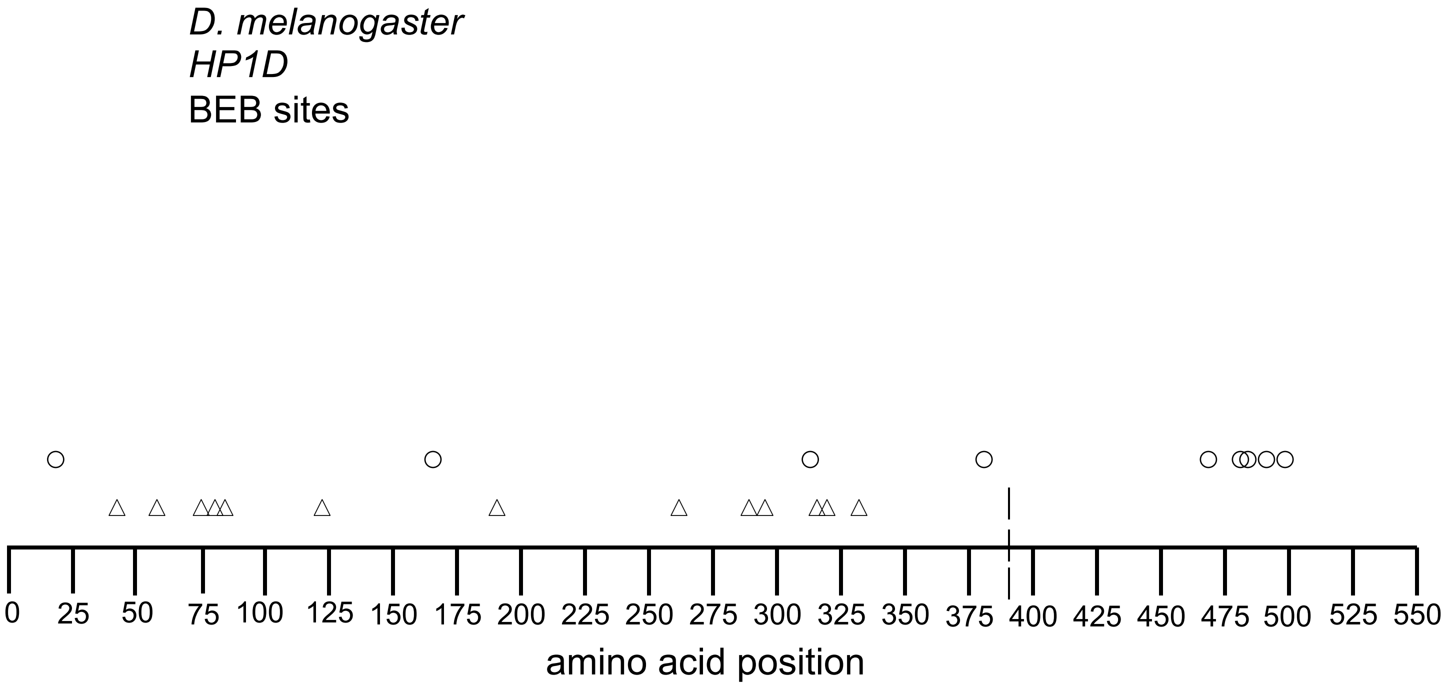

Supplementary Figure 4. Distribution of positively selected sites in the *HP1D* gene in the *D. melanogaster lineage* identified using the BEB method. Triangles indicate the location of positively selected sites, circles indicate the location of polymorphisms. The vertical dashed line represents the separation of exon 1 and exon 2.

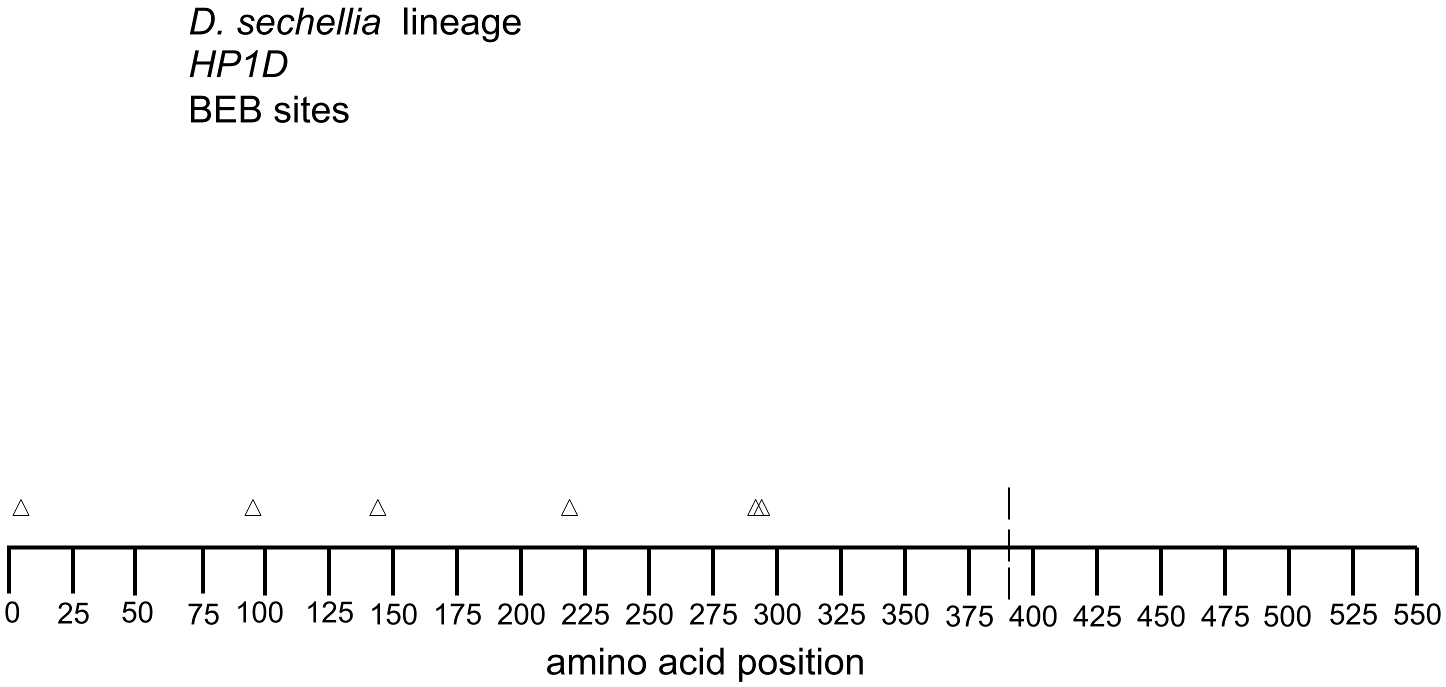

Supplementary Figure 5. Distribution of positively selected sites in the *HP1D* gene in the *D. sechellia lineage* identified using the BEB method. Triangles indicate the location of positively selected sites. The vertical dashed line represents the separation of exon 1 and exon 2.

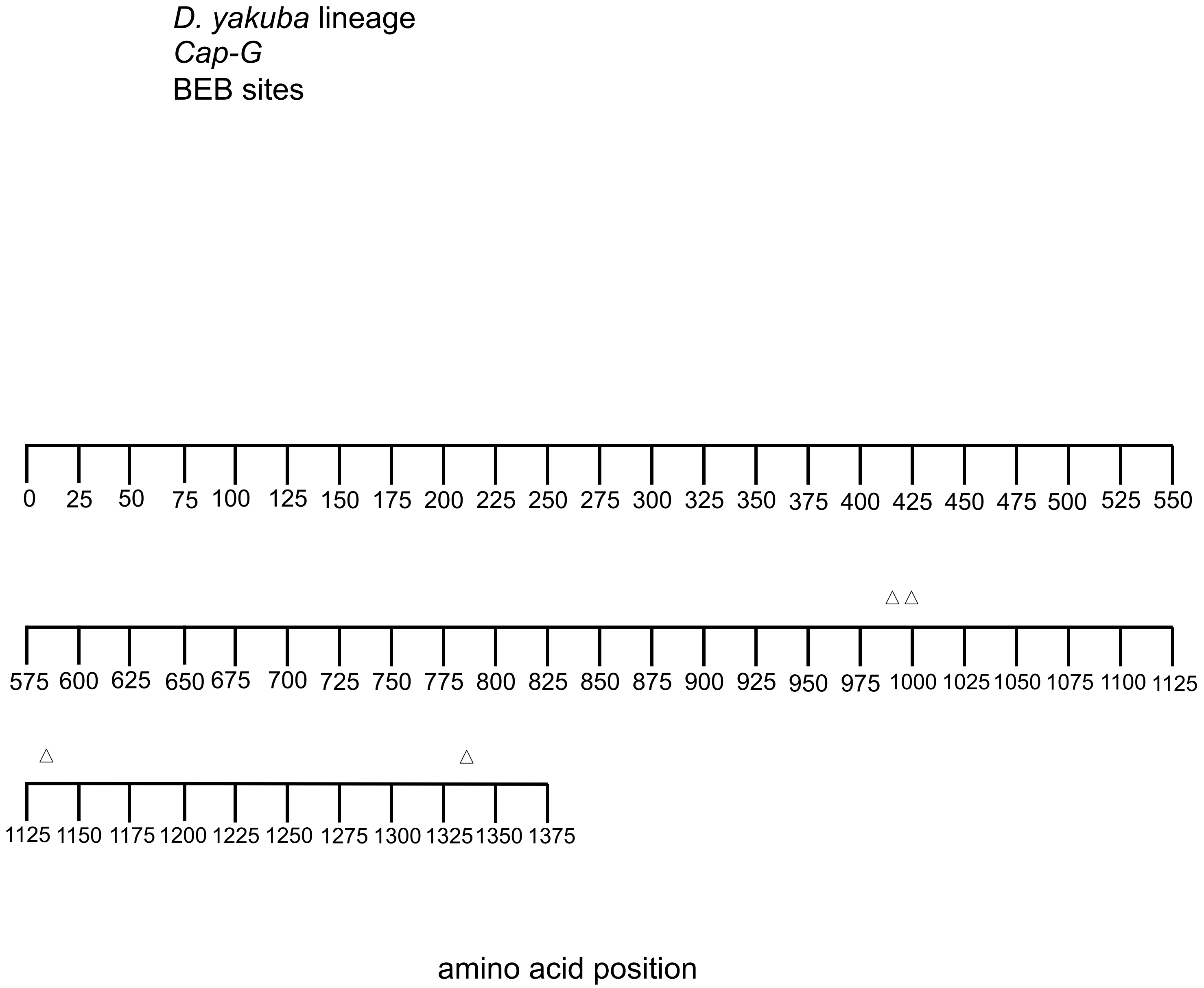

Supplementary Figure 6. Distribution of positively selected sites in the *Cap-G* gene in the *D. yakuba lineage* identified using the BEB method. Triangles indicate the location of positively selected sites.

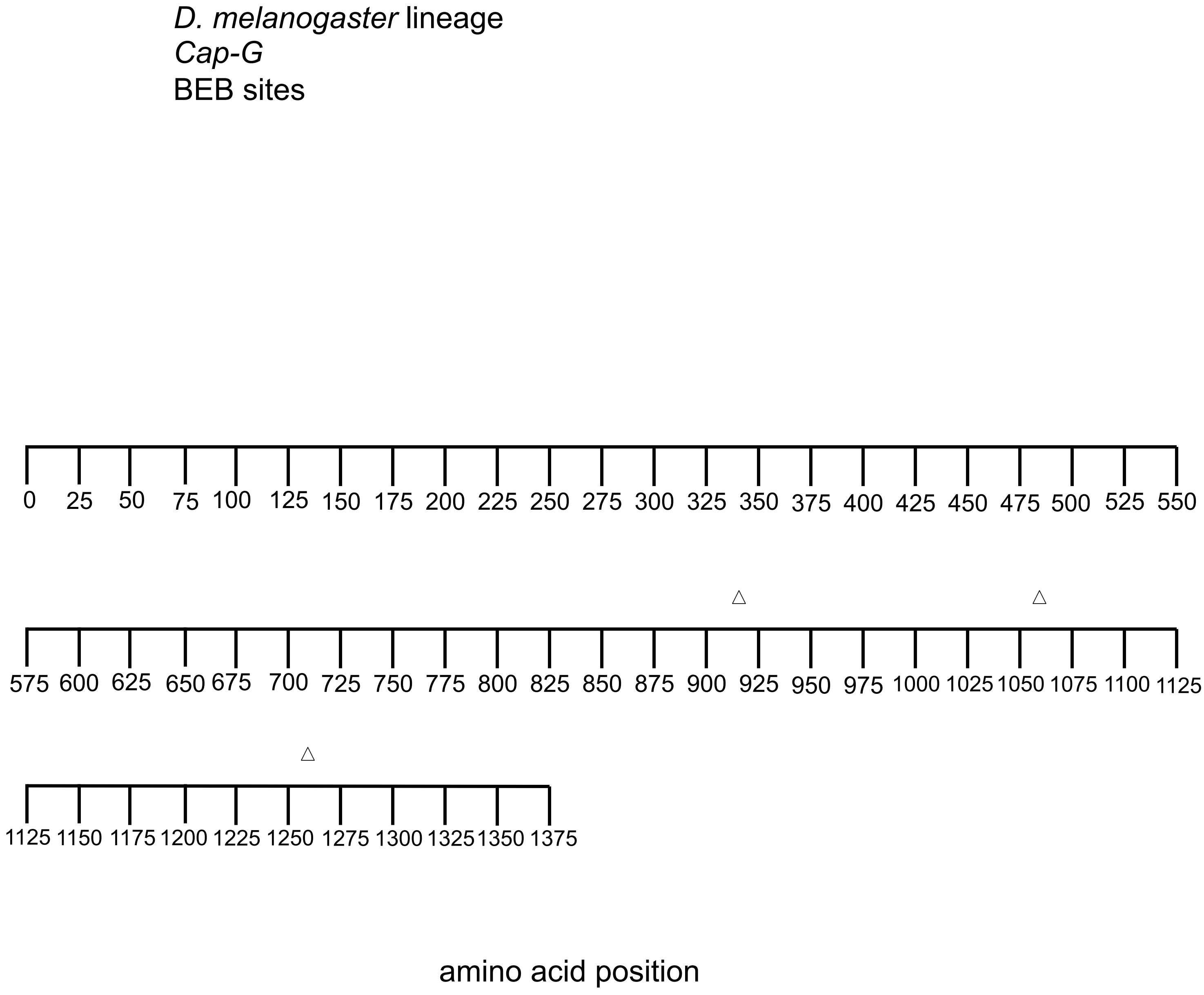

Supplementary Figure 7. Distribution of positively selected sites in the *Cap-G* gene in the *D. melanogaster lineage* identified using the BEB method. Triangles indicate the location of positively selected sites.

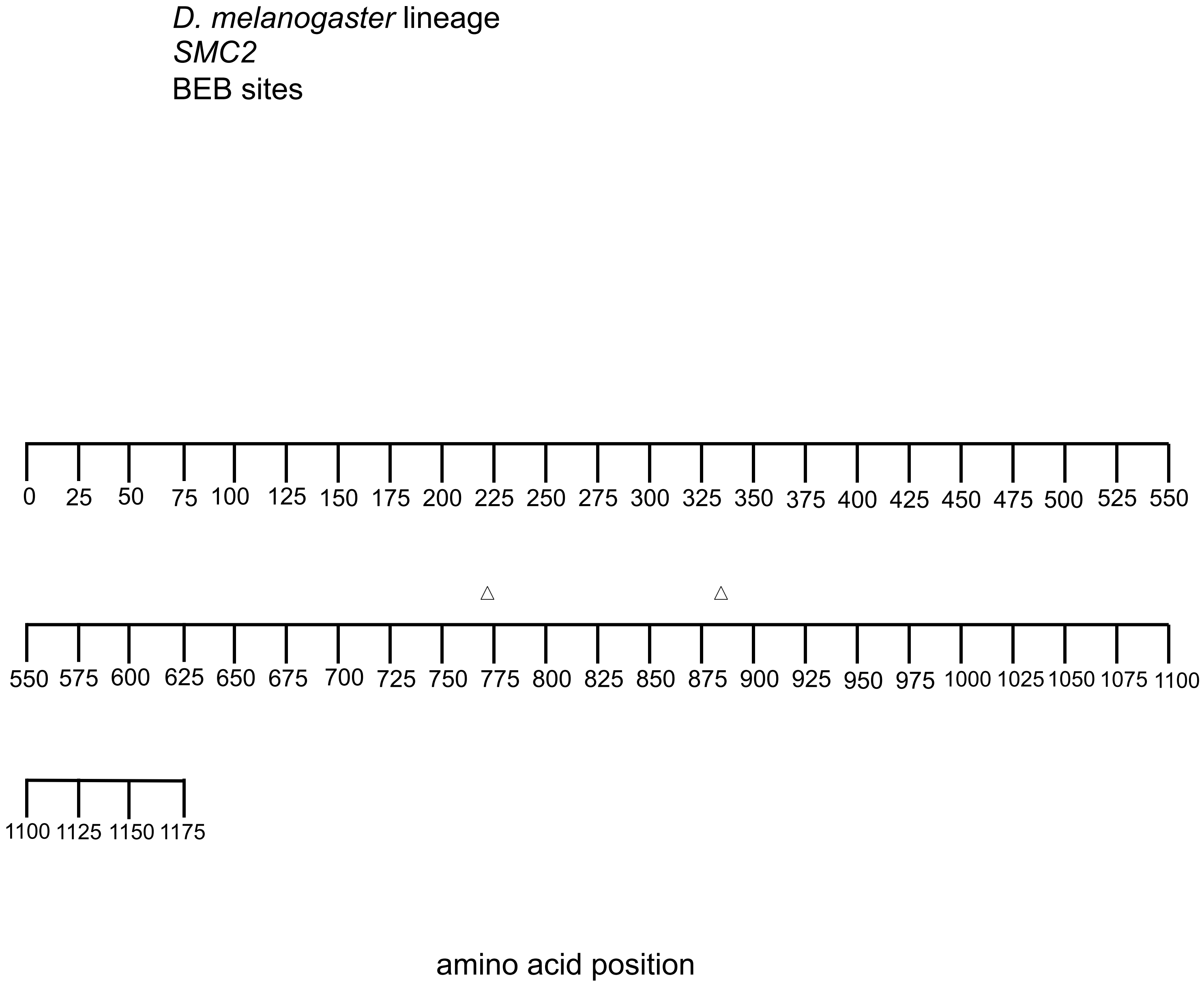

Supplementary Figure 8. Distribution of positively selected sites in the *SMC2* gene in the *D. melanogaster lineage* identified using the BEB method. Triangles indicate the location of positively selected sites.

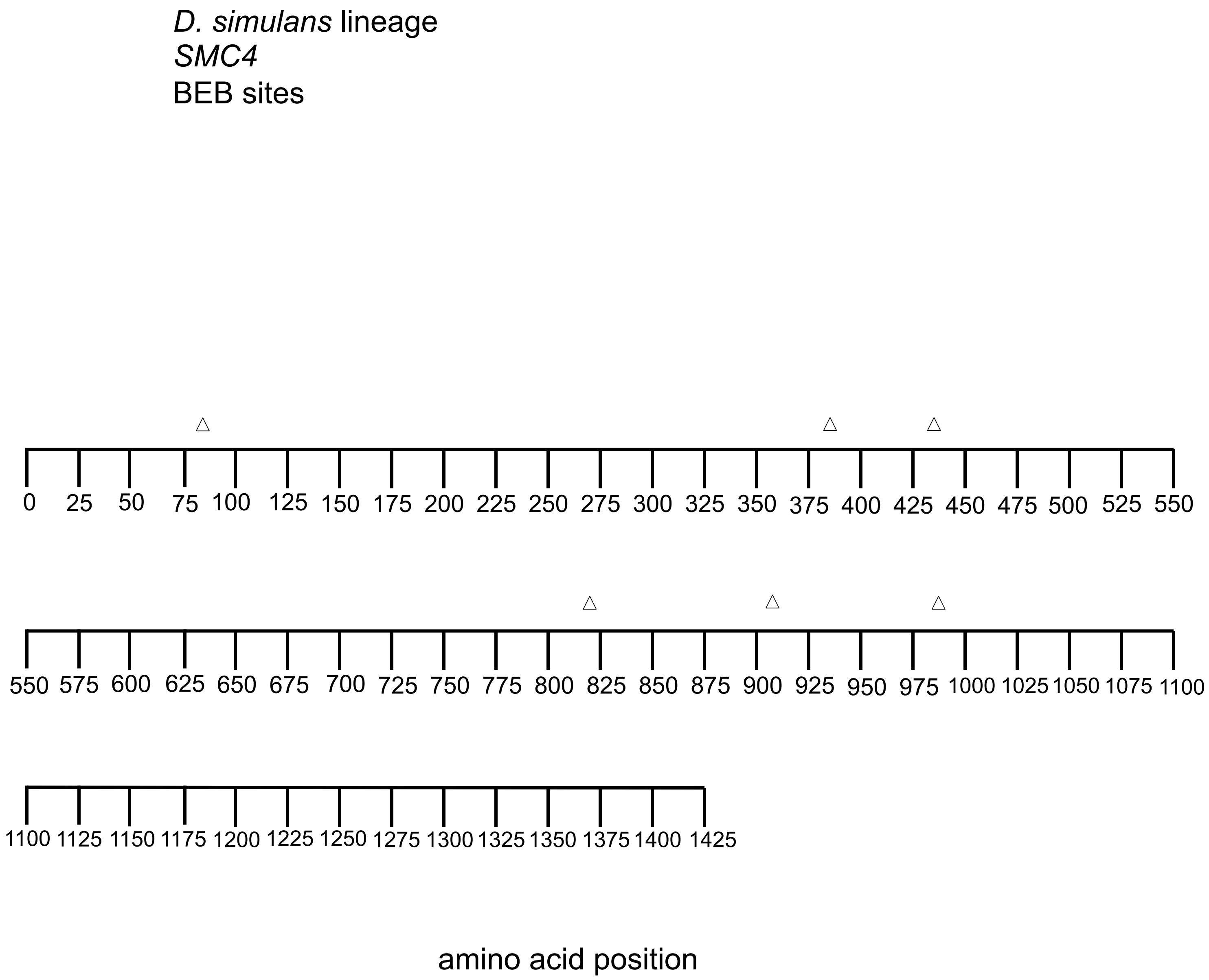

Supplementary Figure 9. Distribution of positively selected sites in the *SMC4* gene in the *D. simulans lineage* identified using the BEB method. Triangles indicate the location of positively selected sites.
